# Opposing Epigenetic Signatures in Human Sperm by Intake of Fast Food Versus Healthy Food

**DOI:** 10.3389/fendo.2021.625204

**Published:** 2021-04-23

**Authors:** Adelheid Soubry, Susan K. Murphy, Greet Vansant, Yang He, Thomas M. Price, Cathrine Hoyo

**Affiliations:** ^1^ Epidemiology Research Center, Department of Public Health and Primary Care, Faculty of Medicine, KU Leuven—University, Leuven, Belgium; ^2^ Department of Obstetrics and Gynecology, Division of Reproductive Sciences, Duke University Medical Center, Durham, NC, United States; ^3^ Duke University School of Medicine, Duke Cancer Institute, Durham, NC, United States; ^4^ Department of Obstetrics and Gynecology, Division of Reproductive Endocrinology and Infertility, Duke University Medical Center, Durham, NC, United States; ^5^ Department of Biological Sciences, Center for Human Health and the Environment, North Carolina State University, Raleigh, NC, United States

**Keywords:** nutrition, sperm, imprinted genes, POHaD, TIEGER study, high fat food

## Abstract

Animal experiments have demonstrated that diets high in fats create a harmful environment for developing sperm cells, contributing to impaired reproductive health and induced risk for chronic diseases in the next generation. Changes at the level of the epigenome have been suggested to underlie these observations. Human data are limited to verify this hypothesis. While we earlier demonstrated a link between male obesity and DNA methylation changes at imprinted genes in mature sperm cells and newborns, it is currently unknown if -or how- a paternal eating pattern (related to obesity) is related to indices for epigenetic inheritance. We here aim to examine a yet unexplored link between consumption of healthy (rich in vitamins and fibers) or unhealthy (“fast”) foods and methylation at imprint regulatory regions in DNA of sperm. We obtained semen and data from 67 men, as part of a North Carolina-based study: The Influence of the Environment on Gametic Epigenetic Reprogramming (TIEGER) study. Dietary data included intake of fruits/nuts, vegetables/soups, whole grain bread, meat, seafood/fish, and fatty or processed food items. Multiple regression models were used to explore the association between dietary habits and clinical sperm parameters as well as DNA methylation levels, quantified using bisulfite pyrosequencing at 12 differentially methylated regions (DMRs) of the following imprinted genes: *GRB10*, *IGF2*, *H19*, *MEG3*, *NDN*, *NNAT*, *PEG1/MEST, PEG3*, *PLAGL1*, *SNRPN*, and *SGCE/PEG10*. After adjusting for age, obesity status and recruitment method, we found that Total Motile Count (TMC) was significantly higher if men consumed fruits/nuts (β=+6.9, SE=1.9, p=0.0005) and vegetables (β=+5.4, SE=1.9, p=0.006), whereas consumption of fries was associated with lower TMC (β=-20.2, SE=8.7, p=0.024). Semen volume was also higher if vegetables or fruits/nuts were frequently consumed (β=+0.06, SE=0.03, p=0.03). Similarly, our sperm epigenetic analyses showed opposing associations for healthy *versus* fast food items. Frequent consumption of fries was related to a higher chance of sperm being methylated at the *MEG3-IG* CpG4 site (OR=1.073, 95%CI: 1.035-1.112), and high consumption of vegetables was associated with a lower risk of DNA methylation at the *NNAT* CpG3 site (OR=0.941, 95%CI: 0.914-0.968). These results remained significant after adjusting for multiple testing. We conclude that dietary habits are linked to sperm epigenetic outcomes. If carried into the next generation paternal unhealthy dietary patterns may result in adverse metabolic conditions and increased risk for chronic diseases in offspring.

## Introduction

Environmental exposures that do not change DNA sequences can alter gene expression in developing germ cells through epigenetic mechanisms. Consequently, fertility may be effected or offspring may inherit these modifications and suffer from phenotypes or chronic diseases their parents never developed. It is known from animal models that chronic consumption of high-energy diets (1, 2), saturated fat (3), or trans fatty acids (4) impairs spermatogenesis. While high fat food constitutes an environmental stress that leads to metabolic diseases and defects of the male reproductive system, it may also cause adverse pregnancy outcomes and growth retardation in offspring (5, 6). A potential explanation could be that long-term dietary exposure to high fat diet (HFD) changes the sperm epigenome; and if inherited, it may induce new phenotypes in the offspring. Notably, a similar mechanism has been reported for other exposures such as from environmental toxins (7). In male rats, Ng et al. showed that HFD not only resulted in offspring with impaired insulin secretion and glucose intolerance, but DNA methylation was also altered in a key pancreatic islet gene, *Il13ra2* (8). A link through the male germ cell epigenome was revealed six years later (9). The investigators injected sperm transfer RNA-derived small RNAs (tsRNAs) from HFD males into normal zygotes and generated offspring with altered gene expression of pathways important in metabolic processes (9). This effect was perpetuated through the second-generation offspring (10). Others also reported that paternal diet affects cardiovascular wellbeing in both the F1 and F2 generation of mice (11).

Human data on potential effects of paternal high-energy or high fat dietary consumption are limited. However, comparable results were found in studies exploring related exposures, such as male obesity. A meta-analysis by Campbell et al., representing epidemiological studies of 115,000 male participants, showed that men with high Body Mass Index (BMI) were more likely to experience infertility problems, sperm morphology defects and DNA fragmentation (12). Similarly, a meta-analysis by Sermondade et al. showed that obese men were at higher risk for oligozoospermia or azoospermia, compared to men of normal weight (13). Only a few studies in humans have attempted to link obesity-related effects in sperm to the epigenome (14, 15). As was shown through animal experiments this could ultimately lead to higher incidences of chronic diseases in the offspring, particularly if they inherit these “paternally-modified” epigenetic signatures. To our knowledge, no human data are available on epigenetic effects in sperm from dietary factors related to obesity such as high-caloric intake or high-fat diets. To fill this gap, on the one side we explore food items from a typical fast food diet -with high substances of saturated fats, trans fats, sugars and salt (16)- for their potential effects on DNA methylation in sperm. On the other side, we explore potential effects from food items that refer to a healthy diet, such as vegetables, whole grains, fruits and nuts (rich in vitamins, fibers and unsaturated fats). Our study includes imprinted genes important in early embryonic growth: *Growth factor Receptor-Bound protein 10* (*GRB10*), *H19*, *Insulin-like Growth Factor 2* (*IGF2*), *Maternally Expressed Gene 3* (*MEG3*), *Necdin* (*NDN*), *Neuronatin* (*NNAT*), *Paternally Expressed Gene 1*/*Mesoderm Specific Transcript* (*PEG1/MEST*), *Paternally Expressed Gene 3* (*PEG3*), *Pleiomorphic Adenoma Gene-Like 1* (*PLAGL1*), *Epsilon Sarcoglycan*/*Paternally Expressed Gene 10* (*SGCE*/*PEG10*), and *Small Nuclear Ribonucleoprotein Polypeptide N* (*SNRPN*).

## Materials and Methods

### Participants

Male volunteers were recruited as part of The Influence of the Environment on Gametic Epigenetic Reprogramming (TIEGER) study. The study description has been published earlier (15, 17). In brief, this North Carolina-based epidemiological study was designed to explore potential epigenetic influences from the environment in sperm from young volunteers recruited from the fertility clinic and elsewhere (e.g. through advertisements in local e-newspapers). Environmental factors studied in the TIEGER population included obesity (15), indoor toxins (17), but also consumption of fast food or high fat food items and healthier foods such as fruits and vegetables, presented in this study. Between May 2012 and November 2013 we collected sperm and dietary records from 67 eligible young Caucasian men (aged 18-35 years old) in the area of Durham, NC (U.S.A.). Eligibility criteria included: being able to produce a semen sample as directed (at the clinic following an abstinence of 3 to 10 days), non-smoker, no personal history of cancer, no vasectomy or other procedures that may cause infertility.

### Data Collection

At recruitment, BMI was measured by the study nurse and a questionnaire was completed by the participants, soliciting information on socio-demographic factors, including level of education, marital status (married or living with partner *versus* single, divorced or widow; indicating if the participant was living alone or not), number of biological children, and occupation. A questionnaire regarding food and portions consumed “yesterday” and over the “last 7 days” was also included. This was obtained through a short list of items used to rank subjects by their dietary habits (healthy or unhealthy) rather than their precise levels of intake. Note, our 7-day recall was used to take into account food items that are not consumed on a daily basis (such as fish). This approach is similar to a previously suggested and validated “screeners” assessment by others (18, 19). The following foods were questioned per serving: fruits and nuts (per cup of fresh fruit; per 1/2 cup of dried fruit, per 1/4 cup of nuts), vegetables, lettuce and vegetable soups (per cup of vegetables/soup; per 2 cups of leafy greens), whole grain bread, flakes or similar (per slice of bread, roll, or cup of flakes); meats (per oz), seafood and fish (per oz), burger or hot-dog (per bun), pizza (per slice), and fries (per 3 oz or 1 small fast food size). Questions are added in **Supplementary Table 1**. All but one participant completed the survey.

### Semen Collection and Clinical Analyses

Semen was collected, processed and stored as described earlier (15). In brief, the World Health Organization’s Laboratory Manual for the Examination and Processing of Human Semen 5th edition was referenced for normal values (20). Semen was analyzed for standard clinical parameters after liquefaction, no later than 60 minutes from collection. Study parameters included motility (%) and Total Motile Count (TMC) (10^6^); the latter has been calculated as (sperm density × ejaculate volume × total motility) ÷ 100. After completion of the clinical sperm analyses, the samples were subjected to two-step ISolate-gradient centrifugation (Irvine Scientific) to select a motile population enriched in normal morphology. This colloidal silica gradient, consisting of a 90% lower layer and 50% upper layer, is prepared by sequentially adding 1.5 ml of each layer to a 15 ml polystyrene conical tube. The sperm sample was pipetted on top of the upper layer and centrifuged at 200x g for 15 minutes. The gradient solution was removed, and the pelleted sperm was stored in an ultracold freezer at -80°C for subsequent DNA methylation analyses.

### DNA Methylation Measurements at Imprinted Genes

Genomic DNA was extracted from 67 sperm samples using Puregene Reagents (Qiagen; Valencia, CA) (**Supplementary Figure 1** **).** Genomic DNA (800 ng) was treated with sodium bisulfite using the EZ DNA Methylation Kit (Zymo Research; Irvine, CA). After bisulfite treatment, DNA (~40 ng, assuming complete recovery) was amplified by PCR in a 25 µl reaction volume. Primer sequences and PCR conditions used for the differentially methylated regions (DMRs) were reported previously (15, 21, 22). Briefly, the 5’ end of one primer of each PCR primer pair was conjugated to biotin to facilitate post-PCR retention of one strand with streptavidin beads. Using the Pyrosequencing WorkStation, the single strand was isolated and then underwent pyrosequencing using a PyroMark Q96 MD pyrosequencing instrument (Qiagen). The following DMRs were tested: *GRB10* (6 CpG sites, chr 7p12.2), *NDN* (6 CpG sites, chr 15q11.2), *NNAT* (3 CpG sites, chr 20q.11.2), *PLAGL1* (6 CpG sites, chr 6q24)*, SGCE/PEG10* (6 CpG sites, chr 7q21.3), *SNRPN* (4 CpG sites, chr 15q11.2), *PEG1/MEST* (4 CpG sites, chr 7q21.3) and *PEG3* (10 CpG sites, chr 19q13.43). Additionally, the region upstream from *IGF2* exon 3, including three CpG dinucleotides (chr 11p15.5), was tested (23); as well as the DMR for *H19*, including four CpG sites (chr 11 p15.5). Finally, two DMRs for the *DLK1/MEG3* imprinted domain were analyzed; they consist of *MEG3-IG* (4 CpG sites) and *MEG3* (8 CpG sites) at chr 14q32.2. A graphical representation of the analyzed DMRs is shown in **Supplementary Figure 2**. Assay validation data and the results of sensitivity tests for pyrosequencing have been previously described. We have shown that pyrosequencing can distinguish as little as 0.5% differences when methylation levels are low (15, 21, 22). Notably, although frequently used, a limitation of using bisulfite pyrosequencing is that it is not possible to distinguish between 5mC and 5hmC.

### Statistical Analyses

Pearson’s correlation tests were used to evaluate relationships between daily and weekly intake of foods, and to measure potential correlations between frequencies of different foods. T-tests were used to evaluate relationships between variables such as age and obese/overweight, and age and patient status. A chi-square test was used to evaluate whether patient status was related to being obese/overweight. Multiple regression models were used to evaluate the relationships between the exposure of interest and sperm outcomes. Raw and adjusted models were compared. We further accounted for multiple testing of several outcomes (sperm characteristics or DMRs) through an adaptive False Discovery Rate (FDR) procedure (24, 25). Sensitivity tests were performed as explained below.

Beta regression models were performed to analyze DNA methylated percentages (26, 27). Variables were divided by 100, which scaled our data in an interval of [0,1]. Hence, DNA methylation values are represented as proportions ranging from 0 (if CpG sites are unmethylated) to 1 (if the CpG site is methylated). A logit link function was used; hence, each *β*-coefficient represents the estimated change on a logit scale, meaning that exp(*β*) equals the odds ratio (OR) when the exposures changes by one portion, keeping other regressors constant. In **Tables 5** and **6** we include ORs for ease of interpreting food-methylation associations. Sensitivity analyses using subgroups of non-patients (n=48), daily food variables instead of weekly reported items, and a grouped exposure analysis (where we defined “fast foods” as the sum of burgers, hot-dogs, pizzas and fries) were repeated and compared. We further repeated our analyses using a robust regression approach to reduce the potential influence of outliers in response and predictor space (28). All analyses were performed using SAS software, version 9.4 of the SAS System for Windows.

## Ethics

The TIEGER Study was performed with the approval of the Duke University Institutional Review Board (protocol Pro00036645). Written informed consent was obtained from all participants for the use of their biological specimens and questionnaire data.

## Results

### Characteristics of Study Participants, Associated Clinical Sperm Parameters, and Daily *versus* Weekly Dietary Patterns

Socio-demographic data of the study population are shown in **Table 1**. Our study population represents young men. The upper age limit to participate in this study was 35. Nearly 70% were younger than 30 years old. Most men had no children (87%). One-third were categorized as overweight or obese. Having a BMI of 25 or more was strongly associated with age (p=0.001). Patients recruited from clinic (n=19; 28.4%) were more likely to be overweight or obese compared to volunteers recruited through advertisements (p<0.001). While most men did not show abnormal clinical sperm parameters at the time of sample collection, patients recruited from the fertility clinic were more likely to have abnormal sperm motility (p=0.007) and a low sperm volume (p=0.042). The few numbers of men with abnormal *versus* normal clinical sperm characteristics are shown in **Table 1**. Only 3 men (4.6%) were categorized as having oligozoospermia, and asthenozoospermia was detected in 19.7%. Overall, mean values were as follows: motility: 52.2% (SD: 13.1), sperm concentration: 76.4x10 ^6^ (SD: 49.6x10 ^6^, semen volume: 3.4 ml (SD: 1.4), and TMC: 129.6x10 ^6^ (SD: 104.5x10^6^). The average time of abstinence in our participants was 4.38 ± 1.41 days.

**Table 1:** Socio-demographics and clinical sperm data of TIEGER study participants.

TIEGER participants (n = 67)		n*	%
**Age**	18-24 years	27	40.3
	25-29 years	19	28.4
	30-35 years	21	31.3
**Marital status**	Single/divorced/widow	36	54.5
	Married/Living with partner	30	45.5
**Fathered at least 1 child**	No	58	86.6
	Yes	9	13.4
**Highest degree of education**	High School	6	10.7
	Some college or college degree	32	57.1
	Graduate	18	32.1
**Obesity status**	normal weight (18 ≤ BMI < 25)	44	66.7
	overweight or obese (25 ≤ BMI)	23	33.3
**Patient at Fertility Clinic**	No	48	71.6
	Yes	19	28.4
**Sperm total motile count** **(TMC)**	≤39x10^6^ (abnormal)	12	18.2
	>39x10^6^ (normal)	54	81.8
**Sperm motility**	<40% (asthenozoospermia)	13	19.7
	≥40% (normal)	53	80.3
**Sperm concentration**	<15 x10^6^ (oligozoospermia)	3	4.6
	≥15 x10^6^ (normal)	62	95.4

*If the sum was not 67, data were missing, and percentage was calculated on known data.

Our study population consumed a mean of one serving size of fruits or nuts per day. The reported consumption of vegetables, soups or salads was 1.2 (SD: 1.1) servings per day. The mean intake of servings of meat was 1.6 (SD: 1.3) servings per day, and 1.5 (SD: 2.2) servings of fish per week. We further verified whether the reported set of food items consumed in one day was correlated to the number of food items men reported in one week (“last 7 days”). Strong positive correlations were measured between weekly and daily intake (**Table 2**). Because some foods items are less frequently consumed (such as fish and seafood), we continued our statistical analyses on the reported weekly intake of all food items measured and the list of food items consumed in the last 24 hours was used in an additional sensitivity analysis. Distribution of food items consumed per week are shown in **Table 3**. A correlation analysis (**Supplementary Table 3**) showed that consumption of fruits or nuts was related to consumption of greens (such as vegetables, lettuce or soup) (*ρ* =0.807, p<0.0001). A significant negative but weaker association was found between consumption of greens (vegetables, lettuce or soup) and pizza (*ρ*= -0.285, p= 0.024), and a negative (but not significant) association was seen between consumption of fruits or nuts and pizza (*ρ*= -0.206, p= 0.10). A significant positive correlation was found between consumption of pizza and fries (*ρ* = 0.40, p= 0.001). A graphical representation of all correlations between consumption of the food items studied is shown in **Supplementary Figure 3**. In a search for potential correlations between dietary patterns and obesity we measured a trend between low consumption of whole grain bread and obesity or overweight (p=0.104).

**Table 2:** Reported daily foods *versus* weekly foods.

Food item	Day - Week correlation (rho)	p-value
**Fruits or nuts**	0.697	<0.0000001
**Vegetables, lettuce or soup**	0.751	<0.0000001
**Whole grain bread or flakes**	0.856	<0.0000001
**Meat**	0.712	<0.0000001
**Seafood or fish**	0.594	0.0000002
**Burger or hotdog**	0.773	<0.0000001
**Pizza**	0.651	<0.0000001
**Fries**	0.536	0.0000058

Pearson’s correlation tests between foods consumed ‘yesterday’ and over ‘the last 7 days’.

**Table 3:** Descriptives of food items consumed by TIEGER participants in one week.

Food item	mean	SD	min.	max.
**Fruits or nuts**	7.18	6.39	0	28
**Vegetables, lettuce or soup**	8.46	6.48	0	35
**Whole grain bread or flakes**	12.99	10.86	0	48
**Meat**	11.13	6.60	0	30
**Seafood or fish**	1.46	2.16	0	14
**Burger or hotdog**	1.40	1.99	0	14
**Pizza**	1.99	2.38	0	8
**Fries**	1.27	1.45	0	7

### Associations Between Dietary Pattern and Clinical Sperm Outcomes

A crude association test, as well as our analyses adjusting for potential confounders and multiple testing, suggested that both consumption of fruits/nuts and vegetables are positively associated with semen volume (β= +0.062, SE= 0.03, p= 0.028, for fruits and nuts; β= +0.063, SE= 0.026, p= 0.020, for vegetables) and TMC (β=+6.95, SE=1.89, p=0.0005, for fruits and nuts; β=+5.40, SE=1.90, p=0.006, for vegetables) (**Table 4**). For example, this means that an extra portion of fruits/nuts per week increases the total number of motile sperm cells with 7.10 ^6^ per ejaculate. We further found a negative association between frequency of consumption of fries and TMC, which was significant after adjusting for age, BMI, and patient status (β=-20.21, SE=8.74, p=0.024) (**Table 4**). Other food items did not show significant associations with TMC or semen volume (see **Supplementary Table 3**).

**Table 4:** Associations between dietary items and semen xharacteristics.

Food item	Sperm factor	Adjusted for age, BMI, and patient status	
		β	(SE; p-value)
**Fruits/nuts**	**TMC**	**+6.95**	**(1.89; 0.0005)**
**Vegetables**		**+5.40**	**(1.90; 0.006)**
**Whole grains**		+1.10	1.24; 0.378
**Meat**		+3.18	1.96; 0.111
**Fish**		+8.91	6.20; 0.156
**Burger**		+1.40	7.00; 0.843
**Pizza**		-6.731	5.534; 0.229
**Fries**		**-20.21**	**(8.74; 0.024)**
**Fruits/nuts**	**Motility**	+0.279	0.255; 0.278
**Vegetables**		+0.154	0.246; 0.532
**Whole grains**		+0.087	0.154; 0.572
**Meat**		+0.031	0.247; 0.900
**Fish**		+ 1.295	0.764; 0.095
**Burger**		-0.858	0.852; 0.318
**Pizza**		+0.366	0.686; 0.596
**Fries**		-0.479	1.118; 0.670
**Fruits/nuts**	**Concentration**	+1.460	0.967; 0.136
**Vegetables**		+1.213	0.931; 0.198
**Whole grains**		+ 0.312	0.588; 0.598
**Meat**		+0.534	0.944; 0.574
**Fish**		-2.397	2.932; 0.417
**Burger**		+4.572	3.226; 0.162
**Pizza**		-2.861	2.615; 0.278
**Fries**		-8.293	4.164; 0.051
**Fruits/nuts**	**Volume**	**+ 0.062**	**0.028; 0.028**
**Vegetables**		**+0.063**	**0.026; 0.020**
**Whole grains**		+0.008	0.017; 0.656
**Meat**		+0.005	0.028; 0.865
**Fish**		+0.152	0.081; 0.065
**Burger**		-0.001	0.097; 0.990
**Pizza**		-0.094	0.076; 0.224
**Fries**		-0.040	0.125; 0.749

Results of linear regression models are represented for all potential associations between food items and semen characteristics (n = 67). In bold: significant associations (p < 0.05).

### DNA Methylation in Sperm in Relation to Frequency of Dietary Intake of Specific Food Items

The number of CpGs tested at each DMR of the genes of interest and the calculated mean DNA percentage of methylation at each gene in sperm cells are shown in **Supplementary Table 2**. Nine DMRs were largely unmethylated (averages of CpG sites: *GRB10* (1.82%), *MEG3* (1.78%), *PEG1/MEST* (1.63%), *NDN* (1.52%), *NNAT* (1.87%), *PEG3* (1.52%), *SGCE/PEG10* (3.13%), *SNRPN* (1.60%)) and 3 DMRs were nearly fully methylated (*H19* (88.06%), *IGF2* (93.92%), *MEG3-IG* (80.00%)). We evaluated potential associations between frequency of food intake the week before sample collection and DNA methylation outcomes at each CpG. Significant results are shown in **Table 5** and the list of all associations tested are shown in **Supplementary Table 4**. In brief, after taking into account potential confounding by age, BMI, and patient status, increased consumption of pizza was positively associated with DNA methylation at *IGF2* CpG2 (β=0.046, SE=0.020; p=0.026) and at *MEG3-IG* CpG 2 (β=0.024, SE=0.009, p=0.009). This translates to 4.8% (95%CI: 0.6%-9.1%) increased risk for DNA methylation at the *IGF2* CpG3 site for an additional slice of pizza (to the mean), and 2.4% (95%CI: 0.6-4.2%) increased risk for DNA methylation at the *MEG3-IG* CpG2. Increased consumption of fries was associated with higher DNA methylation at *IGF2* CpG3 (β=+0.152, SE=0.073, p=0.043), *MEG3-IG* CpG2 (β=+0.034, SE=0.014, p=0.021) and *MEG3-IG* CpG4 (β=+0.070, SE=0.018, p=0.0002); meaning that increased risk for DNA methylation was 16.4% (95%CI: 0.5%-0.34%) at *IGF2* CpG3, 3.4% (95%CI: 0.5%-6.4%) at *MEG3-IG* CpG2, and 7.3% (95%CI: 3.5%-11.2%) at *MEG3-IG* CpG4. The later finding was still significant after accounting for the number of genomic sites tested.

**Table 5:** Associations between diet and DNA methylation levels at CpG sites of imprinted genes.

Food item	DMR CpG	Associations	Adjusted for age, BMI, patient status	
**Pizza**	*IGF2* CpG2*	β (SE; p)	+0.046	(0.020; 0.026)
		OR (95%CI)	1.048	(1.006 - 1.091)
	*MEG3-IG* CpG2	β (SE; p)	+0.024	(0.009; 0.009)
		OR (95%CI)	1.024	(1.006 - 1.042)
	*MEG3-IG* CpG4	β (SE; p)	+0.023	(0.012; 0.063)
		OR (95%CI)	1.023	(0.999 - 1.048)
**Fries**	*IGF2* CpG3*	β (SE; p)	+0.152	(0.073; 0.043)
		OR (95%CI)	1.164	(1.005 - 1.348)
	*MEG3-IG* CpG2	β (SE; p)	+0.034	(0.014; 0.021)
		OR (95%CI)	1.034	(1.005 - 1.064)
	*MEG3-IG* CpG4*	β (SE; p)	**+0.070**	**(0.018; 0.0002)**
		OR (95%CI)	**1.073**	**(1.035 - 1.112)**
**Whole grain bread**	*IGF2* CpG2*	β (SE; p)	-0.010	(0.004; 0.021)
		OR (95%CI)	0.990	(0.982 - 0.998)
	*IGF2* CpG3	β (SE; p)	-0.019	(0.010; 0.065)
		OR (95%CI)	0.981	(0.962 - 1.001)
	*MEG3-IG* CpG3*	β (SE; p)	-0.003	(0.001; 0.026)
		OR (95%CI)	0.997	(0.995 - 1.000)
**Vegetables**	*NNAT* CpG3*	β (SE; p)	**-0.061**	**(0.014; <0.0001)**
		OR (95%CI)	**0.941**	**(0.914 - 0.968)**

Beta regression models are shown if the adjusted models were significant (p < 0.05) (TIEGER data, n = 67). Beta-coefficients are provided in log-scale. The estimated Odds ratios (ORs) represent the odds that DNA methylation will deviate from the mean given one additional portion of food has been consumed in one week. In bold are the results that remain significant after a stringent correction for multiple testing taking into account multiple genomic sites. *Represent sites where associations remained significant after reducing the potential influence of outliers. Items that did not show any significant results are: fruits, nuts, meats, hot-dogs, burgers, fish, and seafood.

Healthy food items, such as whole grain bread consumption was associated with decreased DNA methylation at *IGF2* CpG2 (β =-0.010, SE=0.004, p=0.021; OR=0.990, 95%CI: 0.982-0.998) and at *MEG3-IG* CpG3 (β =-0.003, SE=0.001, p=0.026; OR=0.997, 95%CI: 0.995-1.000). Consumption of vegetables was related to a decrease in DNA methylation at *NNAT* CpG3 (β=-0.061, SE=0.014, p<0.0001; OR=0.941, 95%CI: 0.914-0.968); this remained significant after FDR correction for multiple testing.

No significant relationships were found between CpG methylation and food items such as fruits, nuts, all types of meat, burgers, hot-dogs, seafood, or fish.

We further performed several additional sensitivity analyses. Although reduction of our sample size (e.g., using non-patients only) reduced precision of our estimates, the overall results did not change (data not shown). This suggests that our findings were not unduly influenced by patient-status or by the use of a food survey by week, instead of a 24-hour questionnaire. Next, we performed additional tests to exclude potential influences from outliers, and found similar results.

Because CpGs of one DMR are correlated, we also considered the mean methylation percentage of all CpGs per DMR for each gene; our findings did not differ. Significant results are represented in **Table 6** and all associations tested are shown in **Supplementary Table 5**. In brief, after correcting of potential confounders, consumption of pizza was still associated with increased DNA methylation at the *IGF2* DMR (β=+0.032, SE=0.015, p=0.036). Eating fries was related to DNA methylation at the *IGF2* DMR (β=+0.053, SE=0.024, p=0.033) and *MEG3-IG* DMR (β=+0.019, SE=0.010, p=0.048). An opposite effect was seen for an increased number of portions of vegetables at the *NNAT* DMR (β=-0.026, SE=0.011, p=0.029); meaning that eating vegetables increases the number of unmethylated sperm cells at the *NNAT* DMR.

**Table 6:** Associations between diet and the mean DNA methylation level at imprinted genes.

Food item	Gene	Associations	Adjusted for age, BMI and patient status	
**Pizza**	*IGF2*	β (SE; p)	+0.032	(0.015; 0.036)
		OR (95%CI)	1.032	(1.002 - 1.064)
**Fries**	*IGF2*	β (SE; p)	+0.053	(0.024; 0.033)
		OR (95%CI)	1.054	(1.005 - 1.106)
	*MEG3-IG*	β (SE; p)	+0.019	(0.010; 0.048)
		OR (95%CI)	1.019	(1.000 - 1.039)
**Vegetables**	*NNAT*	β (SE; p)	-0.026	(0.011; 0.029)
		OR (95%CI)	0.974	(0.952 - 0.997)

The mean of the CpGs for each of the 12 DMRs were considered (TIEGER data, n = 67). Beta regression models were fitted for each DMR. Results are shown if models were significant (p < 0.05). Beta-coefficients are provided in log-scale. The estimated Odds ratios (ORs) represent the odds that DNA methylation deviates from the mean given one additional portion of food has been consumed in one week. All associations remained significant after reducing the potential influence of outliers.

## Discussion

In this study we evaluated associations between the reported frequency of consumption of common food items, sperm motility and DNA methylation profiles in sperm of men aged ≤35 years. We found that participants who frequently consumed unhealthy or fat food items, such as pizza and fries, had more sperm cells in their ejaculate that were fully methylated at the DMRs of *IGF2* and *MEG3-IG*. Especially the latter remained significant after adjusting for multiple testing of several DMRs. Hence, these regions have a tendency to be closer to the theoretically expected patterns (being 100% methylated) in sperm, if men ate unhealthy foods. While we cannot explain this potential paradoxical result, earlier observations were comparable. A positive association between high BMI and DNA methylation at the *MEG3-IG* DMR was found in the same population of men (15); and, a similar magnitude of differential methylation at *MEG3-IG* was also measured in offspring of obese fathers in the NEST cohort (29). Our data further suggest that consumption of whole grain bread was associated with less DNA methylation at *IGF2* and *MEG3-IG*. Interestingly, while the majority of the imprinted gene DMRs studied are located within CpG islands, the *IGF2* DMR is an exception (30). This DMR was originally defined as a region for which altered methylation was associated with colon cancer risk (31). This DMR has also been extensively studied in humans for its vulnerability to environmental exposures. For instance, Shen et al. reported that early life exposure to famine and high total cholesterol levels in adulthood were associated with elevated DNA methylation at these CpG sites (32). Our data further show that consumption of other healthy items, such as vegetables, soups, and salads, was linked to lower DNA methylation % at the *NNAT* DMR. Levels of DNA methylation changes were small, in the order of a few to 5% per portion of foods consumed per week. Notably, similar small effect changes of DNA methylation have been observed in other epidemiological studies where poor health outcomes in offspring were linked to nutritionally poor diet around conception (33, 34). Another interesting observation in the current study is the potential positive influence of vegetables, fruits and nuts on clinical sperm characteristics and a negative effect from frequently eating fries. It has been well documented that a prudent diet, characterized by fruits, vegetables, whole grains and other low fat or low cholesterol food items, is positively associated with sperm count, while fast food, rich in processed and fried foods, is inversely related to semen quality (35, 36).

A possible explanation for dietary influences on the sperm epigenome could be the fact that some dietary components, such as fries and pizza, are a source of high-carbs and high fats. This may disrupt testicular metabolism by means of excessive Reactive Oxygen Species (ROS) production. It has been shown that in addition to DNA damage through oxidation of DNA molecules, ROS signaling plays an important role in epigenetic processes such as DNA methylation and histone modification (37). Although the exact mechanism is not known, this suggests that an unhealthy food pattern may cause DNA hypermethylation through ROS (38). Animal data provide evidence for involvement of epigenetic changes in the male germ line after consumption of high-fat diet (8, 39–41). However, the exact underlying molecular mechanism has yet to be discovered.

In addition, fries and pizza ingredients (such as toppings) contain trans fatty acids. These partially hydrogenated oils (PHOs) are also known as trans fats. Since June 2018, the FDA has banned trans fats from processed and packaged foods in the US, because of their contribution to cardiovascular diseases and other chronic disorders (42). However, during the time of recruitment into our study (2013-2014), people were still significantly exposed to trans fats. For instance, the trans fat content of a large bundled fast food meal represents 50% to 75% of the these recommendation in 2014 (16). It remains a challenge to completely eliminate PHOs from the food chain, especially on a global level. A review by Rato et al. indicates that accumulation of trans-fatty acids in the testes (from dietary intake) disrupts *de novo* lipogenesis in Sertoli cells, causing dysfunctional spermatogenesis (2). While more research is needed to explore potential effects of PHOs on the sperm epigenome, it would be of interest to repeat our study in a more recently recruited population of men after the official ban of trans fats in 2018 to learn about the potential beneficial effects of this intervention. Next, it is also possible that a high concentration of acrylamide in fries, formed when foods are cooked at high temperatures, alters the sperm epigenome (43). This carcinogenic substance has recently been added to the list of chemicals that causes transgenerational damage. Male rodents exposed to acrylamide before conception produce offspring with altered protein levels and increased levels of DNA damage in their germ cells (44). These results raise concerns if translatable to humans.

Another unexplored hypothesis is a potential effect from Bisphenol A or Phthalates on the sperm epigenome. The NHANES study in the US population showed that fast food consumption is an important source of exposure to both chemicals (45). Given endocrine disruptors and other environmental chemicals have been linked to changes in the sperm epigenome (41, 46–48), a potential influence from frequent consumption of fast food should be further explored in future studies.

Finally, fast food consumption and obesity are highly associated (49). We earlier showed that paternal obesity could be linked to DNA methylation changes in newborns (29, 50). A recent study by Noor et al. in 429 father-mother-infant triads indicated that such changes might be persistent. They showed an association between paternal obesity and persistent changes in the epigenome in offspring at the ages of 3 and 7 years (51). We hypothesize that other related exposures, such as preconceptional dietary patterns of the father, also influence offspring’s epigenetic signatures and health.

A strength of the current study is the ability to compare our fast-food related results with our earlier published results on male obesity from the same population (15). The current approach shows that after adjusting for obesity, DNA methylation was still altered at DMRs of imprinted genes if frequency of eating fast food was high. This suggests that the underlying factor in earlier reported obesity related epigenetic alterations might be related to food type instead of obesity status. This finding is encouraging, especially in the context of possible interventions to reverse these effects in sperm and ultimately also in offspring. An interesting finding is the fact that our data suggests a beneficial effect of eating vegetables on the *NNAT* gene in sperm. This knowledge could for example help reduce harmful effects from environmental exposures on DNA methylation of *NNAT* (52).

Most epigenetic studies collect data on mother-child pairs, with less focus on paternal exposures. Maternal nutrition and/or food supplementation is known to influence *in utero* development. Pioneering work from Waterland and Jirtle in the Agouti mouse model showed that gene programming *via* the epigenome can be modified through dietary modifications during early development (53, 54). Therefore, exploring epigenetic mechanisms in germ cells and in offspring samples holds great promise to improve our understanding about induced changes in development by nutritional and other environmental factors. A recent report regarding a multi-center pregnancy cohort in Australia shows that frequent consumption of fast foods and infrequent intake of fruit delays time to pregnancy (55). However, they did not interrogate the epigenome and only maternal dietary habits were questioned. Taking into account the fact that imprinted genes are important in early embryo growth, our results indicate that a healthy diet and lifestyle of the male partner before pregnancy may be as important as a healthy diet and lifestyle before and during pregnancy in the future mother.

A weakness of our study is the small sample size and the cross-sectional nature of the study design; hence, a causal relationship cannot be confirmed. In addition, our analysis was restricted to Caucasian subjects due to unknown influence of race/ethnicity on the epigenome. We recommend repeating our study in a wider range of the population. Although self-reported, our food survey was completed by nearly all participants. We did not include long food frequency questionnaires or dietary records. Instead, we focused on specific items from the food pyramid to estimate dietary food patterns in our subjects. Taking into account the dietary guidelines provided by the USDA (56), few participants met the daily recommendations on intake of vegetables (which is 3 to 4 portions per day for males between 18 and 30 years old). Most men reported only 1 portion per day (or 7 per week). A similar observation can be made about fruit consumption in our study population. Notably, the USDA categorizes nuts together with protein foods, while we added nuts together with fruit in our survey. Hence, the mean intake of fruits measured in the TIEGER population of one portion per day is most likely overestimated. Because few men consumed fruits, we cannot exclude a potential beneficial effect of high fruit intake on the sperm epigenome. Next, by using a two-group categorization of fruits and nuts it is not possible to determine if the beneficial effects on sperm parameters were related to fruit-related nutrients (such as specific vitamins) or nutrients originating from nuts (such as unsaturated fats). Other items, such as meat or fish consumption were in accordance with the daily USDA recommendations. Our list of food items did not completely cover the largest section of the food pyramid: carbohydrates, e.g. pasta, white bread, potatoes, rice, etc., were not included. However, it was our focus to explore healthy habits, such as consumption of whole grain food items, and unhealthy food habits, such as consumption of fast foods or meals high in saturated fats (from which consumption is recommended to keep as low as 10% of the daily calories). Notably, the saturated fat content of the large-sized meal from fast-food chains in the US in 2013 was 61% to 80% (depending on the fast-food chain); hence, 6 to 8 times higher than the recommended 10% upper limit (16). Food items rich in cheese (such as pizza) have been defined as the top-1 source of saturated fats in the US population diet (57). In future studies, we propose that it will be important to use a more detailed dietary questionnaire including the different dietary fats, lipids, and vitamins; preferentially taken over a longer time-period. This would help to better understand the influence of fats and micronutrients on the sperm epigenome. We hypothesize that nutrient-induced epigenetic marks can be maintained from sperm cell to embryo, affecting health of the next generation. Despite the limitations of the current pilot study our data supports part of this hypothesis and opens new perspectives on the role of paternal life style in preconceptional health.

Ultimately, our results may also help identifying a set of biomarkers measuring the impact from environmental exposures. The current and our former studies indicate that the same parameters in sperm are susceptible to set of environmental exposures (including nutrition, obesity and endocrine disruptors) (17, 29, 50). This is in line with other reports where specific DMRs have been suggested as biosensors for evaluating earlier exposures (58, 59).

## Conclusions

The current study suggests a yet unreported epigenetic effect of diet on human sperm cells. Although we cannot explain the exact cellular mechanisms underlying the associations we observed, it is striking that methylation effect changes were similar for unhealthy fatty or processed foods, and opposite associations were measured when men consumed healthy dietary items. Because DNA methylation changes at these imprinted genes have been linked with adverse metabolic conditions and an increased risk for chronic diseases in offspring, we stress the need for more research on the paternal role in the transmission of acquired environmental messages from father to child. Similarly, future research that focuses on how lifestyle and dietary interventions change the human sperm epigenome remains to be studied. Our data fits our earlier new concept of the Paternal Origins of Health and Disease (POHaD) (60), where the role of the father has been suggested in disease development of children. If better understood, this knowledge could be applied in public health. If dietary changes positively (or adversely) shape the human sperm epigenetic profile, programming in the offspring can subsequently be influenced.

## Data Availability Statement

The datasets that support the results of this study are available from the corresponding author upon reasonable request.

## Ethics Statement

The studies involving human participants were reviewed and approved by Duke University Institutional Review Board (protocol Pro00036645). The patients/participants provided their written informed consent to participate in this study.

## Author Contributions

AS initiated the research question, study hypothesis and statistical analyses. AS and CH developed the epidemiological study design. SM developed the experimental study designs of the TIEGER study. TP oversaw patient recruitment and semen analyses. SM oversaw laboratory DNA methylation analyses. YH performed supplementary statistical analyses. GV contributed to the interpretation of the short list of food items as the exposure of interest. All authors contributed to the article and approved the submitted version.

## Funding

This work was supported by a *Health and the Environment* award from the Duke Cancer Institute and the Duke Nicholas School of the Environment, and a research grant from KU Leuven University [OT/14/109].

## Conflict of Interest

The authors declare that the research was conducted in the absence of any commercial or financial relationships that could be construed as a potential conflict of interest.
