# Supplementary material for: Opposing Epigenetic Signatures in Human Sperm by Intake of Fast Food Versus Healthy Food
Source: Front Endocrinol (Lausanne). 2021 Apr 23;12:625204. doi: 10.3389/fendo.2021.625204 (PMC8103543; doi:10.3389/fendo.2021.625204)
Supplement: Supplementary file 1 [file DataSheet_1.pdf]

frontiers

Supplementary Material

Supplementary Figure 1: Flow Diagram of the TIEGER Study Population

Participant flow chart of the TIEGER study

81 men presented themselves at the clinic to join the study

↓

75 men met the study criteria

↓

69 men signed the informed consent

↓

67 men had enough sperm to perform DNA methylation and statistical analyses

Legend:
Healthy male participants were recruited as part of The Influence of the Environment on Gametic Epigenetic Reprogramming Male (TIEGER) cross-sectional study. Participants were recruited at the fertility clinic and through internet advertisements and flyers distributed within the city limits of Durham, NC, from May 2012 to November 2013. Eligibility criteria included: self-reported Caucasian ethnicity/race, non-smoking, no personal history of cancer, no vasectomy or other procedures that could cause infertility, and 18-35 years of age. A total of eighty-one men contacted the clinic. Six men were excluded because they were non-Caucasian. Six men declined participation prior to informed consent, resulting in sixty-nine Caucasian men completing the study. In two men we failed to obtain enough sperm cells because of azoospermia, hence DNA methylation analyses was obtained for 67 of 69 Caucasian participants. Data design and collection have also been discussed earlier [14,16].
