# Supplementary material for: Opposing Epigenetic Signatures in Human Sperm by Intake of Fast Food Versus Healthy Food
Source: Front Endocrinol (Lausanne). 2021 Apr 23;12:625204. doi: 10.3389/fendo.2021.625204 (PMC8103543; doi:10.3389/fendo.2021.625204)

Supplementary Figure 2 (A-F): Graphical representation of DMRs of imprinted genes of interest

A: *GRB10* and *NNAT*; B: *IGF2* and *H19*; C: *MEG3*, D: *MEST* and *PEG3*; E: *NDN* and *SNRPN*; F: *PLAGL1* and *SGCE/PEG10*. Color codes and symbols used on the genome representing: CpG islands (green ovals), ICR CTCF binding sites (orange ovals), coding regions (tall pink boxes), noncoding regions (short pink boxes), antisense (purple boxes), single arrows (transcription start sites).

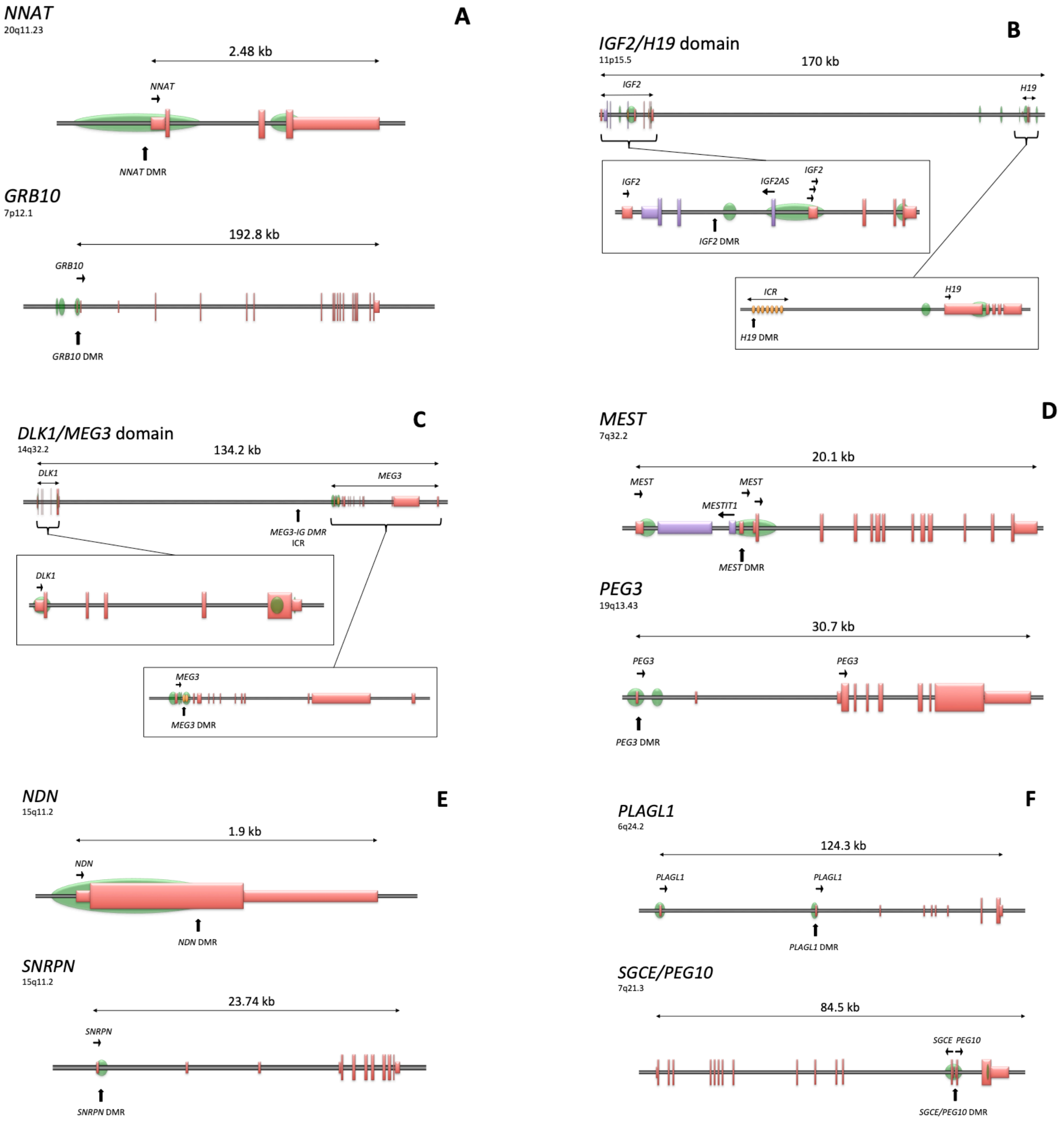

Supplement: Supplementary file 2 [file DataSheet_2.pdf]
