# Supplementary figures and images for: Opposing Epigenetic Signatures in Human Sperm by Intake of Fast Food Versus Healthy Food
Source: Front Endocrinol (Lausanne). 2021 Apr 23;12:625204. doi: 10.3389/fendo.2021.625204 (PMC8103543; doi:10.3389/fendo.2021.625204)

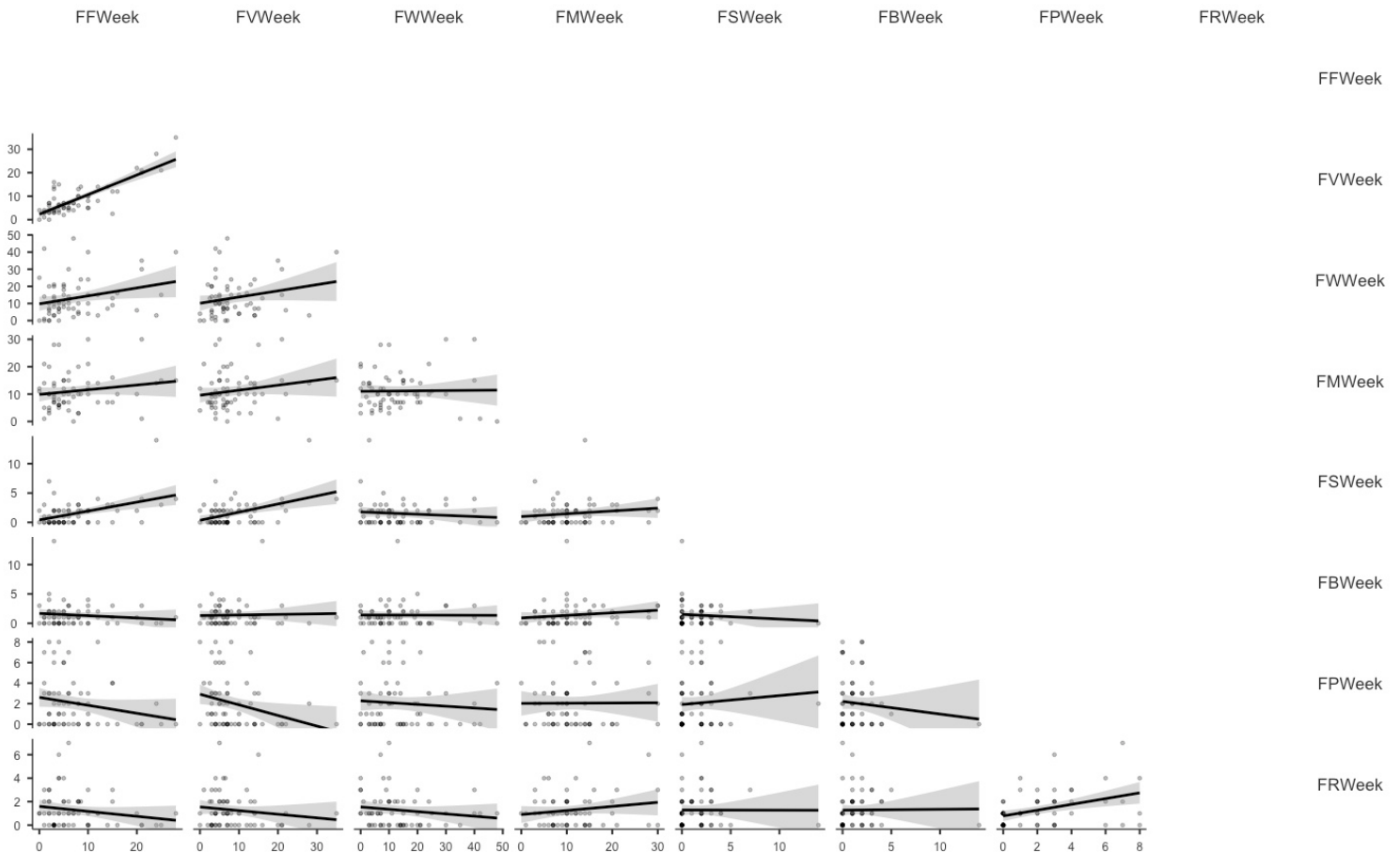

Supplement: Supplementary file 3 [file DataSheet_3.pdf]
