# Supplementary material for: Opposing Epigenetic Signatures in Human Sperm by Intake of Fast Food Versus Healthy Food
Source: Front Endocrinol (Lausanne). 2021 Apr 23;12:625204. doi: 10.3389/fendo.2021.625204 (PMC8103543; doi:10.3389/fendo.2021.625204)
Supplement: Supplementary file 4 [file DataSheet_4.pdf]

frontiers

Supplementary Material

Supplementary Table 1: Food consumption survey in TIEGER Participants

The following questions were asked by a trained study nurse and recorded in the form of a table:
1. How many portions of **Fruits or nuts** did you eat over the **last 7 days?** 1 portion = 1 cup of fresh fruit, or 1/2 cup of dried fruit, or 1/4 cup of nuts.<br>2. How many portions of **Vegetables, lettuce, or vegetable soup** did you eat over the **last 7 days?** 1 portion = 1 cup of soup or veggies, or 2 cups of leafy greens.<br>3. How many portions of **Whole grain bread or flakes** did you eat over the **last 7 days?** 1 portion = 1 cup of flakes, 1 slice of bread, 1 roll.<br>4. How many portions of **Meat (beef, pork, turkey, sausage, chicken,...)** did you eat over **The last 7 days?** 1 portion = 4 oz (the size of a deck of cards).<br>5. How many portions of **Seafood or Fish** did you eat over the **last 7 days?** 1 portion = 4 oz (the size of a deck of cards).<br>6. How many portions of **Burger or Hot-dog** did you eat over the **last 7 days?** 1 portion = meat plus one bun.<br>7. How many portions of **Pizza** did you eat over the **last 7 days?** 1 portion = 1 slice.<br>8. How many portions of **Fries** did you eat over the **last 7 days?** 1 portion = 3 oz or a small fast-food size.
1. How many portions of **Fruits or nuts** did you eat **yesterday?** 1 portion = 1 cup of fresh fruit, or 1/2 cup of dried fruit, or 1/4 cup of nuts.<br>2. How many portions of **Vegetables, lettuce, or vegetable soup** did you eat **yesterday?** 1 portion = 1 cup of soup or veggies, or 2 cups of leafy greens.<br>3. How many portions of **Whole grain bread or flakes** did you eat **yesterday?** 1 portion = 1 cup of flakes, 1 slice of bread, 1 roll.<br>4. How many portions of **Meat (beef, pork, turkey, sausage, chicken,...)** did you eat **yesterday?** 1 portion = 4 oz (the size of a deck of cards).<br>5. How many portions of **Seafood or Fish** did you eat **yesterday?** 1 portion = 4 oz (the size of a deck of cards).<br>6. How many portions of **Burger or Hot-dog** did you eat **yesterday?** 1 portion = meat plus one bun.<br>7. How many portions of **Pizza** did you eat **yesterday?** 1 portion = 1 slice.<br>8. How many portions of **Fries** did you eat over the **yesterday?** 1 portion = 3 oz or a small fast-food size.

Questions related to food consumption, asked by a trained nurse at the day of sperm donation.
