# Supplementary material for: Opposing Epigenetic Signatures in Human Sperm by Intake of Fast Food Versus Healthy Food
Source: Front Endocrinol (Lausanne). 2021 Apr 23;12:625204. doi: 10.3389/fendo.2021.625204 (PMC8103543; doi:10.3389/fendo.2021.625204)
Supplement: Supplementary file 5 [file DataSheet_5.pdf]

Supplementary Table 2: Mean DNA Methylation at Imprinted Genes in Sperm from TIEGER Participants

Mean methylation percentages are shown per gene. Methylation profiles at the individual CpG sites have been published earlier (14).

Gene (#CpGs)	*GRB10 (6)*	*H19 (4)*	*IGF2 (3)*	*MEG3 (8)*	*MEG3-IG (4)*	*PEG1 /MEST (4)*	*NDN (6)*	*NNAT (3)*	*PEG3 (10)*	*PLAGL1 (6)*	*SGCE /PEG10 (6)*	*SNRPN (4)*
**mean DNA methylation % (SD)**	1.82 (0.97)	88.06 (2.14)	93.92 (1.73)	1.78 (2.81)	80.00 (1.95)	1.63 (0.52)	1.52 (1.62)	1.87 (1.67)	1.52 (1.41)	4.08 (5.56)	3.13 (3.45)	1.60 (0.94)
