# Supplementary material for: Opposing Epigenetic Signatures in Human Sperm by Intake of Fast Food Versus Healthy Food
Source: Front Endocrinol (Lausanne). 2021 Apr 23;12:625204. doi: 10.3389/fendo.2021.625204 (PMC8103543; doi:10.3389/fendo.2021.625204)
Supplement: Supplementary file 6 [file DataSheet_6.pdf]

Supplementary Table 3: Pearson's correlation test showing associations between food items consumed in one week in the TIEGER participants.

Interrelationship between consumption of food items is represented through Pearson's correlation coefficients. Food items per week are shown as follows: FBWEEK (for burgers), FFWEEK (for fruits/nuts), FMWEEK (for meat), FPWEEK (for pizza), FRWEEK (for fries), FSWEEK (for fish/seafood), FVWEEK (for vegetables/salads/soups), FWWEEK (for whole grain bread/flakes).

		**FFWeek**	**FVWeek**	**FWWeek**	**FMWeek**	**FSWeek**	**FBWeek**	**FPWeek**
**FVWeek**	Pearson's r	0.8071258	—					
	p-value	< .0000001	—					
**FWWeek**	Pearson's r	0.2706261	0.2180580	—				
	p-value	0.0319353	0.0860066	—				
**FMWeek**	Pearson's r	0.1646365	0.1822591	0.0149543	—			
	p-value	0.1972546	0.1528107	0.9073945	—			
**FSWeek**	Pearson's r	0.4428996	0.4202855	-0.0981674	0.1454877	—		
	p-value	0.0002780	0.0006049	0.4440169	0.2552398	—		
**FBWeek**	Pearson's r	-0.1217520	0.0318908	-0.0073280	0.1441325	-0.0855682	—	
	p-value	0.3418238	0.8040420	0.9545451	0.2597410	0.5048995	—	
**FPWeek**	Pearson's r	-0.2063965	-0.2847805	-0.0808287	0.0055176	0.0791758	-0.1040142	—
	p-value	0.1046015	0.0236866	0.5288633	0.9657668	0.5373510	0.4172171	—
**FRWeek**	Pearson's r	-0.1846591	-0.1400506	-0.1475884	0.1570951	-0.0021021	0.0098248	0.4022991
	p-value	0.1473856	0.2736204	0.2483678	0.2188522	0.9869544	0.9390830	0.0010812
