# Supplementary material for: Opposing Epigenetic Signatures in Human Sperm by Intake of Fast Food Versus Healthy Food
Source: Front Endocrinol (Lausanne). 2021 Apr 23;12:625204. doi: 10.3389/fendo.2021.625204 (PMC8103543; doi:10.3389/fendo.2021.625204)
Supplement: Supplementary file 7 [file DataSheet_7.pdf]

# *Supplementary Material*

## Supplementary Table 4: Associations between all Dietary Items and DNA Methylation Levels at CpG Sites at Imprinted Genes

Beta regression models are shown for each CpG site after adjusting for potential confounders (age, BMI, and patient status); TIEGER data (n=67). In bold: significant results (p<0.05). Beta-coefficients are provided in log-scale (outliers were reconsidered). DMRO refers to the DMR of *IGF2*. Food items per week are shown as follows: FBWEEK (for burgers), FFWEEK (for fruits/nuts), FMWEEK (for meat), FPWEEK (for pizza), FRWEEK (for fries), FSWEEK (for fish/seafood), FVWEEK (for vegetables/salads/soups), FWWEEK (for whole grain bread/flakes).

Supplementary Table 4

| CpG | Diet | Beta | SE | p-value |
|---|---|---|---|---|
| H19CBS1_CPG1 | FBWeek | 0.016 | 0.032 | 0.615 |
| H19CBS1_CPG2 | FBWeek | 0.001 | 0.016 | 0.940 |
| H19CBS1_CPG3 | FBWeek | 0.01 | 0.018 | 0.576 |
| H19CBS1_CPG4 | FBWeek | 0.007 | 0.021 | 0.737 |
| MEG3CBS1_CPG1 | FBWeek | -0.026 | 0.065 | 0.691 |
| MEG3CBS1_CPG2 | FBWeek | -0.078 | 0.052 | 0.135 |
| MEG3CBS1_CPG3 | FBWeek | 0.001 | 0.059 | 0.980 |
| MEG3CBS1_CPG4 | FBWeek | 0.025 | 0.038 | 0.515 |
| MEG3CBS1_CPG5 | FBWeek | 0.034 | 0.058 | 0.555 |
| MEG3CBS1_CPG6 | FBWeek | -0.031 | 0.06 | 0.611 |
| MEG3CBS1_CPG7 | FBWeek | 0.037 | 0.044 | 0.392 |
| MEG3CBS1_CPG8 | FBWeek | 0.025 | 0.048 | 0.605 |
| MEG3IG_CPG2 | FBWeek | -0.005 | 0.011 | 0.683 |
| MEG3IG_CPG3 | FBWeek | 0.005 | 0.007 | 0.482 |
| MEG3IG_CPG4 | FBWeek | -0.001 | 0.015 | 0.945 |
| MEG3IG_CPG5 | FBWeek | 0.004 | 0.01 | 0.715 |
| MESTIT1_CPG1 | FBWeek | -0.043 | 0.064 | 0.498 |
| MESTIT1_CPG2 | FBWeek | 0.043 | 0.031 | 0.159 |
| MESTIT1_CPG3 | FBWeek | 0.025 | 0.022 | 0.252 |
| MESTIT1_CPG4 | FBWeek | 0.034 | 0.045 | 0.454 |
| DMRO_CPG1 | FBWeek | -0.003 | 0.011 | 0.804 |
| DMRO_CPG2 | FBWeek | 0.003 | 0.025 | 0.907 |
| DMRO_CPG3 | FBWeek | -0.021 | 0.055 | 0.706 |
| GRB10_CPG1 | FBWeek | -0.054 | 0.065 | 0.406 |
| GRB10_CPG2 | FBWeek | 0.009 | 0.017 | 0.600 |
| GRB10_CPG3 | FBWeek | -0.03 | 0.055 | 0.591 |
| GRB10_CPG4 | FBWeek | -0.102 | 0.063 | 0.104 |
| GRB10_CPG5 | FBWeek | -0.002 | 0.018 | 0.903 |
| GRB10_CPG6 | FBWeek | -0.002 | 0.021 | 0.939 |
| SGCE_CPG1 | FBWeek | 0.004 | 0.039 | 0.917 |
| SGCE_CPG2 | FBWeek | -0.018 | 0.065 | 0.780 |
| SGCE_CPG3 | FBWeek | 0.005 | 0.048 | 0.925 |
| SGCE_CPG4 | FBWeek | -0.022 | 0.041 | 0.585 |
| SGCE_CPG5 | FBWeek | -0.011 | 0.064 | 0.867 |
| SGCE_CPG6 | FBWeek | -0.077 | 0.051 | 0.134 |
| PEG3_CPG1 | FBWeek | -0.035 | 0.042 | 0.412 |
| PEG3_CPG2 | FBWeek | 0.002 | 0.04 | 0.964 |
| PEG3_CPG3 | FBWeek | 0.014 | 0.039 | 0.719 |
| PEG3_CPG4 | FBWeek | 0.012 | 0.052 | 0.817 |
| PEG3_CPG5 | FBWeek | 0.017 | 0.04 | 0.666 |
| PEG3_CPG6 | FBWeek | -0.021 | 0.063 | 0.732 |
| PEG3_CPG7 | FBWeek | -0.001 | 0.034 | 0.972 |

| | | | | |
|---|---|---|---|---|
| PEG3_CPG8 | FBWeek | -0.027 | 0.063 | 0.663 |
| PEG3_CPG9 | FBWeek | 0.004 | 0.03 | 0.906 |
| PEG3_CPG10 | FBWeek | 0.000 | 0.033 | 0.997 |
| PLAGL1_CPG1 | FBWeek | -0.013 | 0.057 | 0.816 |
| PLAGL1_CPG2 | FBWeek | -0.042 | 0.062 | 0.495 |
| PLAGL1_CPG3 | FBWeek | -0.011 | 0.051 | 0.821 |
| PLAGL1_CPG4 | FBWeek | 0.006 | 0.058 | 0.922 |
| PLAGL1_CPG5 | FBWeek | -0.086 | 0.061 | 0.156 |
| PLAGL1_CPG6 | FBWeek | -0.037 | 0.06 | 0.537 |
| NDN_R2bio_CPG1 | FBWeek | 0.013 | 0.039 | 0.744 |
| NDN_R2bio_CPG2 | FBWeek | 0.054 | 0.055 | 0.330 |
| NDN_R2bio_CPG3 | FBWeek | 0.05 | 0.047 | 0.281 |
| NDN_R2bio_CPG4 | FBWeek | 0.021 | 0.047 | 0.652 |
| NDN_R2bio_CPG5 | FBWeek | 0.026 | 0.056 | 0.643 |
| NDN_R2bio_CPG6 | FBWeek | -0.032 | 0.042 | 0.448 |
| SNRPN_CPG1 | FBWeek | -0.004 | 0.023 | 0.854 |
| SNRPN_CPG2 | FBWeek | -0.006 | 0.031 | 0.855 |
| SNRPN_CPG3 | FBWeek | 0.001 | 0.03 | 0.966 |
| SNRPN_CPG4 | FBWeek | -0.021 | 0.063 | 0.743 |
| NNAT_CPG1 | FBWeek | -0.01 | 0.068 | 0.883 |
| NNAT_CPG2 | FBWeek | -0.14 | 0.059 | 0.190 |
| NNAT_CPG3 | FBWeek | -0.035 | 0.043 | 0.413 |
| H19CBS1_CPG1 | FFWeek | 0.001 | 0.008 | 0.875 |
| H19CBS1_CPG2 | FFWeek | 0.000 | 0.005 | 0.982 |
| H19CBS1_CPG3 | FFWeek | -0.002 | 0.005 | 0.750 |
| H19CBS1_CPG4 | FFWeek | -0.002 | 0.006 | 0.679 |
| MEG3CBS1_CPG1 | FFWeek | 0.016 | 0.019 | 0.410 |
| MEG3CBS1_CPG2 | FFWeek | -0.007 | 0.013 | 0.595 |
| MEG3CBS1_CPG3 | FFWeek | 0.011 | 0.017 | 0.543 |
| MEG3CBS1_CPG4 | FFWeek | -0.008 | 0.011 | 0.487 |
| MEG3CBS1_CPG5 | FFWeek | -0.013 | 0.019 | 0.484 |
| MEG3CBS1_CPG6 | FFWeek | -0.034 | 0.018 | 0.054 |
| MEG3CBS1_CPG7 | FFWeek | -0.008 | 0.013 | 0.545 |
| MEG3CBS1_CPG8 | FFWeek | -0.003 | 0.014 | 0.845 |
| MEG3IG_CPG2 | FFWeek | -0.001 | 0.003 | 0.760 |
| MEG3IG_CPG3 | FFWeek | -0.003 | 0.002 | 0.165 |
| MEG3IG_CPG4 | FFWeek | 0.000 | 0.004 | 0.999 |
| MEG3IG_CPG5 | FFWeek | -0.002 | 0.003 | 0.513 |
| MESTIT1_CPG1 | FFWeek | 0.029 | 0.019 | 0.124 |
| MESTIT1_CPG2 | FFWeek | 0.009 | 0.011 | 0.450 |
| MESTIT1_CPG3 | FFWeek | 0.006 | 0.008 | 0.461 |
| MESTIT1_CPG4 | FFWeek | 0.028 | 0.015 | 0.056 |
| DMRO_CPG1 | FFWeek | 0.002 | 0.003 | 0.581 |
| DMRO_CPG2 | FFWeek | -0.006 | 0.007 | 0.385 |
| DMRO_CPG3 | FFWeek | 0.007 | 0.016 | 0.645 |
| GRB10_CPG1 | FFWeek | 0.021 | 0.018 | 0.240 |

| | | | | |
|---|---|---|---|---|
| GRB10_CPG2 | FFWeek | -0.001 | 0.005 | 0.820 |
| GRB10_CPG3 | FFWeek | 0.01 | 0.013 | 0.446 |
| GRB10_CPG4 | FFWeek | 0.028 | 0.017 | 0.088 |
| GRB10_CPG5 | FFWeek | -0.001 | 0.005 | 0.883 |
| GRB10_CPG6 | FFWeek | -0.001 | 0.006 | 0.805 |
| SGCE_CPG1 | FFWeek | -0.009 | 0.011 | 0.406 |
| SGCE_CPG2 | FFWeek | 0.007 | 0.019 | 0.721 |
| SGCE_CPG3 | FFWeek | -0.012 | 0.014 | 0.360 |
| SGCE_CPG4 | FFWeek | -0.011 | 0.011 | 0.294 |
| SGCE_CPG5 | FFWeek | -0.017 | 0.019 | 0.358 |
| SGCE_CPG6 | FFWeek | -0.005 | 0.013 | 0.704 |
| PEG3_CPG1 | FFWeek | 0.003 | 0.011 | 0.792 |
| PEG3_CPG2 | FFWeek | -0.002 | 0.011 | 0.839 |
| PEG3_CPG3 | FFWeek | -0.003 | 0.011 | 0.798 |
| PEG3_CPG4 | FFWeek | -0.002 | 0.016 | 0.893 |
| PEG3_CPG5 | FFWeek | -0.002 | 0.012 | 0.859 |
| PEG3_CPG6 | FFWeek | 0.03 | 0.017 | 0.089 |
| PEG3_CPG7 | FFWeek | -0.002 | 0.01 | 0.836 |
| PEG3_CPG8 | FFWeek | 0.004 | 0.018 | 0.846 |
| PEG3_CPG9 | FFWeek | -0.001 | 0.009 | 0.873 |
| PEG3_CPG10 | FFWeek | 0.005 | 0.009 | 0.595 |
| PLAGL1_CPG1 | FFWeek | -0.006 | 0.017 | 0.699 |
| PLAGL1_CPG2 | FFWeek | 0.003 | 0.018 | 0.864 |
| PLAGL1_CPG3 | FFWeek | -0.012 | 0.014 | 0.411 |
| PLAGL1_CPG4 | FFWeek | -0.003 | 0.017 | 0.876 |
| PLAGL1_CPG5 | FFWeek | 0.004 | 0.017 | 0.813 |
| PLAGL1_CPG6 | FFWeek | 0.001 | 0.017 | 0.954 |
| NDN_R2bio_CPG1 | FFWeek | -0.009 | 0.011 | 0.413 |
| NDN_R2bio_CPG2 | FFWeek | 0.001 | 0.016 | 0.945 |
| NDN_R2bio_CPG3 | FFWeek | -0.003 | 0.014 | 0.853 |
| NDN_R2bio_CPG4 | FFWeek | -0.013 | 0.014 | 0.373 |
| NDN_R2bio_CPG5 | FFWeek | -0.023 | 0.017 | 0.184 |
| NDN_R2bio_CPG6 | FFWeek | 0.006 | 0.012 | 0.633 |
| SNRPN_CPG1 | FFWeek | -0.002 | 0.006 | 0.717 |
| SNRPN_CPG2 | FFWeek | -0.001 | 0.008 | 0.903 |
| SNRPN_CPG3 | FFWeek | -0.007 | 0.008 | 0.433 |
| SNRPN_CPG4 | FFWeek | 0.008 | 0.018 | 0.655 |
| NNAT_CPG1 | FFWeek | -0.008 | 0.021 | 0.696 |
| NNAT_CPG2 | FFWeek | -0.008 | 0.016 | 0.627 |
| NNAT_CPG3 | FFWeek | -0.033 | 0.013 | 0.056 |
| H19CBS1_CPG1 | FMWeek | -0.004 | 0.009 | 0.682 |
| H19CBS1_CPG2 | FMWeek | 0.003 | 0.005 | 0.512 |
| H19CBS1_CPG3 | FMWeek | -0.002 | 0.005 | 0.674 |
| H19CBS1_CPG4 | FMWeek | 0.008 | 0.006 | 0.203 |
| MEG3CBS1_CPG1 | FMWeek | -0.001 | 0.019 | 0.973 |
| MEG3CBS1_CPG2 | FMWeek | -0.003 | 0.013 | 0.833 |

| | | | | |
|---|---|---|---|---|
| MEG3CBS1_CPG3 | FMWeek | -0.016 | 0.017 | 0.345 |
| MEG3CBS1_CPG4 | FMWeek | -0.003 | 0.011 | 0.764 |
| MEG3CBS1_CPG5 | FMWeek | -0.018 | 0.018 | 0.305 |
| MEG3CBS1_CPG6 | FMWeek | -0.004 | 0.017 | 0.826 |
| MEG3CBS1_CPG7 | FMWeek | -0.007 | 0.013 | 0.570 |
| MEG3CBS1_CPG8 | FMWeek | -0.013 | 0.014 | 0.371 |
| MEG3IG_CPG2 | FMWeek | 0.002 | 0.003 | 0.559 |
| MEG3IG_CPG3 | FMWeek | -0.001 | 0.002 | 0.519 |
| MEG3IG_CPG4 | FMWeek | 0.002 | 0.004 | 0.635 |
| MEG3IG_CPG5 | FMWeek | -0.003 | 0.003 | 0.325 |
| MESTIT1_CPG1 | FMWeek | 0.007 | 0.018 | 0.713 |
| MESTIT1_CPG2 | FMWeek | -0.004 | 0.010 | 0.695 |
| MESTIT1_CPG3 | FMWeek | 0.000 | 0.007 | 0.985 |
| MESTIT1_CPG4 | FMWeek | 0.007 | 0.014 | 0.607 |
| DMRO_CPG1 | FMWeek | -0.003 | 0.003 | 0.291 |
| DMRO_CPG2 | FMWeek | 0.001 | 0.007 | 0.966 |
| DMRO_CPG3 | FMWeek | -0.011 | 0.017 | 0.517 |
| GRB10_CPG1 | FMWeek | -0.005 | 0.018 | 0.803 |
| GRB10_CPG2 | FMWeek | -0.001 | 0.005 | 0.875 |
| GRB10_CPG3 | FMWeek | -0.043 | 0.016 | 0.054 |
| GRB10_CPG4 | FMWeek | -0.010 | 0.017 | 0.561 |
| GRB10_CPG5 | FMWeek | -0.006 | 0.005 | 0.226 |
| GRB10_CPG6 | FMWeek | -0.004 | 0.006 | 0.514 |
| SGCE_CPG1 | FMWeek | -0.002 | 0.011 | 0.844 |
| SGCE_CPG2 | FMWeek | 0.009 | 0.019 | 0.641 |
| SGCE_CPG3 | FMWeek | -0.023 | 0.014 | 0.088 |
| SGCE_CPG4 | FMWeek | -0.005 | 0.011 | 0.637 |
| SGCE_CPG5 | FMWeek | 0.005 | 0.018 | 0.794 |
| SGCE_CPG6 | FMWeek | -0.007 | 0.013 | 0.591 |
| PEG3_CPG1 | FMWeek | -0.003 | 0.011 | 0.806 |
| PEG3_CPG2 | FMWeek | -0.007 | 0.011 | 0.510 |
| PEG3_CPG3 | FMWeek | -0.003 | 0.011 | 0.786 |
| PEG3_CPG4 | FMWeek | -0.042 | 0.017 | 0.053 |
| PEG3_CPG5 | FMWeek | 0.003 | 0.011 | 0.788 |
| PEG3_CPG6 | FMWeek | -0.001 | 0.018 | 0.970 |
| PEG3_CPG7 | FMWeek | -0.006 | 0.009 | 0.519 |
| PEG3_CPG8 | FMWeek | 0.006 | 0.018 | 0.728 |
| PEG3_CPG9 | FMWeek | -0.004 | 0.009 | 0.614 |
| PEG3_CPG10 | FMWeek | -0.004 | 0.009 | 0.632 |
| PLAGL1_CPG1 | FMWeek | 0.001 | 0.016 | 0.964 |
| PLAGL1_CPG2 | FMWeek | 0.001 | 0.017 | 0.965 |
| PLAGL1_CPG3 | FMWeek | -0.001 | 0.014 | 0.936 |
| PLAGL1_CPG4 | FMWeek | 0.009 | 0.017 | 0.584 |
| PLAGL1_CPG5 | FMWeek | -0.009 | 0.017 | 0.607 |
| PLAGL1_CPG6 | FMWeek | 0.005 | 0.017 | 0.753 |
| NDN_R2bio_CPG1 | FMWeek | -0.009 | 0.011 | 0.410 |

| | | | | |
|---|---|---|---|---|
| NDN_R2bio_CPG2 | FMWeek | -0.012 | 0.017 | 0.485 |
| NDN_R2bio_CPG3 | FMWeek | -0.012 | 0.014 | 0.466 |
| NDN_R2bio_CPG4 | FMWeek | -0.031 | 0.014 | 0.271 |
| NDN_R2bio_CPG5 | FMWeek | -0.014 | 0.017 | 0.395 |
| NDN_R2bio_CPG6 | FMWeek | -0.014 | 0.013 | 0.290 |
| SNRPN_CPG1 | FMWeek | 0.001 | 0.006 | 0.832 |
| SNRPN_CPG2 | FMWeek | 0.003 | 0.008 | 0.713 |
| SNRPN_CPG3 | FMWeek | -0.013 | 0.008 | 0.115 |
| SNRPN_CPG4 | FMWeek | -0.022 | 0.018 | 0.232 |
| NNAT_CPG1 | FMWeek | -0.013 | 0.02 | 0.524 |
| NNAT_CPG2 | FMWeek | -0.005 | 0.016 | 0.760 |
| NNAT_CPG3 | FMWeek | -0.003 | 0.012 | 0.806 |
| H19CBS1_CPG1 | FPWeek | 0.016 | 0.023 | 0.487 |
| H19CBS1_CPG2 | FPWeek | 0.012 | 0.013 | 0.363 |
| H19CBS1_CPG3 | FPWeek | 0.015 | 0.014 | 0.261 |
| H19CBS1_CPG4 | FPWeek | 0.013 | 0.016 | 0.423 |
| MEG3CBS1_CPG1 | FPWeek | -0.042 | 0.052 | 0.416 |
| MEG3CBS1_CPG2 | FPWeek | -0.018 | 0.036 | 0.624 |
| MEG3CBS1_CPG3 | FPWeek | -0.014 | 0.047 | 0.768 |
| MEG3CBS1_CPG4 | FPWeek | -0.033 | 0.030 | 0.269 |
| MEG3CBS1_CPG5 | FPWeek | -0.009 | 0.049 | 0.851 |
| MEG3CBS1_CPG6 | FPWeek | 0.021 | 0.045 | 0.639 |
| MEG3CBS1_CPG7 | FPWeek | -0.02 | 0.034 | 0.553 |
| MEG3CBS1_CPG8 | FPWeek | -0.032 | 0.038 | 0.397 |
| **MEG3IG_CPG2** | **FPWeek** | **0.024** | **0.009** | **0.009** |
| MEG3IG_CPG3 | FPWeek | -0.002 | 0.006 | 0.710 |
| MEG3IG_CPG4 | FPWeek | 0.023 | 0.012 | 0.056 |
| MEG3IG_CPG5 | FPWeek | -0.007 | 0.007 | 0.340 |
| MESTIT1_CPG1 | FPWeek | -0.031 | 0.053 | 0.562 |
| MESTIT1_CPG2 | FPWeek | -0.04 | 0.032 | 0.212 |
| MESTIT1_CPG3 | FPWeek | 0.001 | 0.021 | 0.954 |
| MESTIT1_CPG4 | FPWeek | -0.045 | 0.044 | 0.302 |
| DMRO_CPG1 | FPWeek | 0.007 | 0.008 | 0.404 |
| **DMRO_CPG2** | **FPWeek** | **0.046** | **0.020** | **0.026** |
| DMRO_CPG3 | FPWeek | 0.07 | 0.047 | 0.135 |
| GRB10_CPG1 | FPWeek | 0.055 | 0.05 | 0.277 |
| GRB10_CPG2 | FPWeek | 0.006 | 0.014 | 0.663 |
| GRB10_CPG3 | FPWeek | 0.034 | 0.037 | 0.366 |
| GRB10_CPG4 | FPWeek | 0.095 | 0.045 | 0.063 |
| GRB10_CPG5 | FPWeek | -0.001 | 0.014 | 0.917 |
| GRB10_CPG6 | FPWeek | -0.003 | 0.016 | 0.843 |
| SGCE_CPG1 | FPWeek | -0.028 | 0.031 | 0.346 |
| SGCE_CPG2 | FPWeek | -0.119 | 0.052 | 0.230 |
| SGCE_CPG3 | FPWeek | -0.003 | 0.036 | 0.930 |
| SGCE_CPG4 | FPWeek | -0.025 | 0.029 | 0.387 |
| SGCE_CPG5 | FPWeek | 0.026 | 0.049 | 0.593 |

| | | | | |
|---|---|---|---|---|
| SGCE_CPG6 | FPWeek | 0.018 | 0.034 | 0.592 |
| PEG3_CPG1 | FPWeek | -0.007 | 0.03 | 0.826 |
| PEG3_CPG2 | FPWeek | -0.017 | 0.03 | 0.568 |
| PEG3_CPG3 | FPWeek | -0.008 | 0.031 | 0.788 |
| PEG3_CPG4 | FPWeek | -0.012 | 0.042 | 0.770 |
| PEG3_CPG5 | FPWeek | -0.025 | 0.032 | 0.431 |
| PEG3_CPG6 | FPWeek | 0.009 | 0.048 | 0.847 |
| PEG3_CPG7 | FPWeek | -0.014 | 0.026 | 0.578 |
| PEG3_CPG8 | FPWeek | 0.000 | 0.049 | 0.997 |
| PEG3_CPG9 | FPWeek | -0.012 | 0.023 | 0.606 |
| PEG3_CPG10 | FPWeek | -0.009 | 0.025 | 0.703 |
| PLAGL1_CPG1 | FPWeek | -0.042 | 0.045 | 0.358 |
| PLAGL1_CPG2 | FPWeek | -0.021 | 0.048 | 0.666 |
| PLAGL1_CPG3 | FPWeek | -0.047 | 0.039 | 0.237 |
| PLAGL1_CPG4 | FPWeek | -0.04 | 0.047 | 0.395 |
| PLAGL1_CPG5 | FPWeek | -0.018 | 0.047 | 0.696 |
| PLAGL1_CPG6 | FPWeek | -0.016 | 0.047 | 0.734 |
| NDN_R2bio_CPG1 | FPWeek | 0.003 | 0.031 | 0.928 |
| NDN_R2bio_CPG2 | FPWeek | -0.043 | 0.047 | 0.361 |
| NDN_R2bio_CPG3 | FPWeek | 0.034 | 0.039 | 0.376 |
| NDN_R2bio_CPG4 | FPWeek | 0.011 | 0.038 | 0.773 |
| NDN_R2bio_CPG5 | FPWeek | -0.005 | 0.048 | 0.914 |
| NDN_R2bio_CPG6 | FPWeek | 0.018 | 0.034 | 0.592 |
| SNRPN_CPG1 | FPWeek | -0.012 | 0.017 | 0.481 |
| SNRPN_CPG2 | FPWeek | -0.014 | 0.023 | 0.525 |
| SNRPN_CPG3 | FPWeek | -0.015 | 0.022 | 0.489 |
| SNRPN_CPG4 | FPWeek | -0.021 | 0.049 | 0.672 |
| NNAT_CPG1 | FPWeek | 0.065 | 0.053 | 0.227 |
| NNAT_CPG2 | FPWeek | -0.043 | 0.042 | 0.301 |
| NNAT_CPG3 | FPWeek | 0.044 | 0.03 | 0.143 |
| H19CBS1_CPG1 | FRWeek | 0.040 | 0.039 | 0.309 |
| H19CBS1_CPG2 | FRWeek | 0.023 | 0.021 | 0.271 |
| H19CBS1_CPG3 | FRWeek | 0.012 | 0.023 | 0.582 |
| H19CBS1_CPG4 | FRWeek | 0.024 | 0.026 | 0.359 |
| MEG3CBS1_CPG1 | FRWeek | -0.008 | 0.085 | 0.925 |
| MEG3CBS1_CPG2 | FRWeek | 0.069 | 0.058 | 0.229 |
| MEG3CBS1_CPG3 | FRWeek | 0.038 | 0.075 | 0.613 |
| MEG3CBS1_CPG4 | FRWeek | 0.015 | 0.049 | 0.757 |
| MEG3CBS1_CPG5 | FRWeek | 0.115 | 0.076 | 0.131 |
| MEG3CBS1_CPG6 | FRWeek | 0.121 | 0.072 | 0.090 |
| MEG3CBS1_CPG7 | FRWeek | 0.023 | 0.057 | 0.684 |
| MEG3CBS1_CPG8 | FRWeek | 0.033 | 0.062 | 0.591 |
| **MEG3IG_CPG2** | **FRWeek** | **0.034** | **0.014** | **0.021** |
| MEG3IG_CPG3 | FRWeek | 0.001 | 0.009 | 0.916 |
| **MEG3IG_CPG4** | **FRWeek** | **0.070** | **0.018** | **0.000** |
| MEG3IG_CPG5 | FRWeek | -0.003 | 0.012 | 0.819 |

| | | | | |
|---|---|---|---|---|
| MESTIT1_CPG1 | FRWeek | 0.078 | 0.083 | 0.347 |
| MESTIT1_CPG2 | FRWeek | -0.072 | 0.051 | 0.156 |
| MESTIT1_CPG3 | FRWeek | 0.048 | 0.03 | 0.116 |
| MESTIT1_CPG4 | FRWeek | -0.081 | 0.07 | 0.247 |
| DMRO_CPG1 | FRWeek | 0.015 | 0.014 | 0.283 |
| DMRO_CPG2 | FRWeek | 0.061 | 0.033 | 0.066 |
| **DMRO_CPG3** | **FRWeek** | **0.152** | **0.073** | **0.043** |
| GRB10_CPG1 | FRWeek | 0.085 | 0.08 | 0.288 |
| GRB10_CPG2 | FRWeek | 0.015 | 0.022 | 0.487 |
| GRB10_CPG3 | FRWeek | -0.011 | 0.065 | 0.868 |
| GRB10_CPG4 | FRWeek | 0.07 | 0.072 | 0.331 |
| GRB10_CPG5 | FRWeek | 0.01 | 0.023 | 0.677 |
| GRB10_CPG6 | FRWeek | 0.009 | 0.026 | 0.730 |
| SGCE_CPG1 | FRWeek | -0.014 | 0.051 | 0.778 |
| SGCE_CPG2 | FRWeek | -0.039 | 0.086 | 0.648 |
| SGCE_CPG3 | FRWeek | 0.039 | 0.059 | 0.507 |
| SGCE_CPG4 | FRWeek | 0.003 | 0.049 | 0.957 |
| SGCE_CPG5 | FRWeek | 0.057 | 0.083 | 0.489 |
| SGCE_CPG6 | FRWeek | -0.013 | 0.06 | 0.826 |
| PEG3_CPG1 | FRWeek | 0.042 | 0.048 | 0.389 |
| PEG3_CPG2 | FRWeek | 0.019 | 0.049 | 0.696 |
| PEG3_CPG3 | FRWeek | 0.032 | 0.049 | 0.513 |
| PEG3_CPG4 | FRWeek | 0.108 | 0.064 | 0.094 |
| PEG3_CPG5 | FRWeek | 0.033 | 0.051 | 0.511 |
| PEG3_CPG6 | FRWeek | 0.122 | 0.077 | 0.115 |
| PEG3_CPG7 | FRWeek | 0.019 | 0.042 | 0.654 |
| PEG3_CPG8 | FRWeek | 0.061 | 0.080 | 0.446 |
| PEG3_CPG9 | FRWeek | 0.007 | 0.039 | 0.853 |
| PEG3_CPG10 | FRWeek | 0.022 | 0.041 | 0.596 |
| PLAGL1_CPG1 | FRWeek | 0.055 | 0.074 | 0.455 |
| PLAGL1_CPG2 | FRWeek | 0.003 | 0.081 | 0.970 |
| PLAGL1_CPG3 | FRWeek | 0.057 | 0.063 | 0.368 |
| PLAGL1_CPG4 | FRWeek | 0.083 | 0.076 | 0.272 |
| PLAGL1_CPG5 | FRWeek | 0.015 | 0.078 | 0.849 |
| PLAGL1_CPG6 | FRWeek | 0.016 | 0.078 | 0.837 |
| NDN_R2bio_CPG1 | FRWeek | 0.038 | 0.051 | 0.451 |
| NDN_R2bio_CPG2 | FRWeek | 0.098 | 0.071 | 0.169 |
| NDN_R2bio_CPG3 | FRWeek | 0.074 | 0.062 | 0.234 |
| NDN_R2bio_CPG4 | FRWeek | 0.056 | 0.062 | 0.366 |
| NDN_R2bio_CPG5 | FRWeek | 0.147 | 0.073 | 0.042 |
| NDN_R2bio_CPG6 | FRWeek | 0.077 | 0.055 | 0.161 |
| SNRPN_CPG1 | FRWeek | 0.016 | 0.029 | 0.584 |
| SNRPN_CPG2 | FRWeek | 0.036 | 0.037 | 0.326 |
| SNRPN_CPG3 | FRWeek | 0.01 | 0.037 | 0.793 |
| SNRPN_CPG4 | FRWeek | 0.113 | 0.079 | 0.151 |
| NNAT_CPG1 | FRWeek | 0.039 | 0.09 | 0.667 |

| | | | | |
|---|---|---|---|---|
| NNAT_CPG2 | FRWeek | -0.001 | 0.069 | 0.992 |
| NNAT_CPG3 | FRWeek | 0.048 | 0.051 | 0.353 |
| H19CBS1_CPG1 | FSWeek | -0.007 | 0.023 | 0.771 |
| H19CBS1_CPG2 | FSWeek | -0.011 | 0.014 | 0.438 |
| H19CBS1_CPG3 | FSWeek | 0.006 | 0.015 | 0.687 |
| H19CBS1_CPG4 | FSWeek | 0.005 | 0.017 | 0.770 |
| MEG3CBS1_CPG1 | FSWeek | 0.017 | 0.057 | 0.770 |
| MEG3CBS1_CPG2 | FSWeek | -0.023 | 0.038 | 0.540 |
| MEG3CBS1_CPG3 | FSWeek | 0.041 | 0.05 | 0.421 |
| MEG3CBS1_CPG4 | FSWeek | -0.031 | 0.033 | 0.352 |
| MEG3CBS1_CPG5 | FSWeek | -0.051 | 0.056 | 0.365 |
| MEG3CBS1_CPG6 | FSWeek | -0.038 | 0.05 | 0.451 |
| MEG3CBS1_CPG7 | FSWeek | -0.034 | 0.038 | 0.361 |
| MEG3CBS1_CPG8 | FSWeek | -0.032 | 0.042 | 0.449 |
| MEG3IG_CPG2 | FSWeek | -0.004 | 0.01 | 0.734 |
| MEG3IG_CPG3 | FSWeek | -0.001 | 0.006 | 0.849 |
| MEG3IG_CPG4 | FSWeek | -0.002 | 0.014 | 0.887 |
| MEG3IG_CPG5 | FSWeek | -0.001 | 0.008 | 0.891 |
| MESTIT1_CPG1 | FSWeek | 0.140 | 0.080 | 0.094 |
| MESTIT1_CPG2 | FSWeek | -0.009 | 0.035 | 0.810 |
| MESTIT1_CPG3 | FSWeek | 0.024 | 0.021 | 0.310 |
| MESTIT1_CPG4 | FSWeek | 0.064 | 0.042 | 0.126 |
| DMRO_CPG1 | FSWeek | 0.005 | 0.009 | 0.550 |
| DMRO_CPG2 | FSWeek | 0.008 | 0.022 | 0.732 |
| DMRO_CPG3 | FSWeek | -0.01 | 0.052 | 0.848 |
| GRB10_CPG1 | FSWeek | -0.078 | 0.058 | 0.179 |
| GRB10_CPG2 | FSWeek | -0.008 | 0.015 | 0.580 |
| GRB10_CPG3 | FSWeek | -0.022 | 0.041 | 0.585 |
| GRB10_CPG4 | FSWeek | -0.004 | 0.055 | 0.939 |
| GRB10_CPG5 | FSWeek | -0.008 | 0.015 | 0.589 |
| GRB10_CPG6 | FSWeek | -0.01 | 0.017 | 0.544 |
| SGCE_CPG1 | FSWeek | -0.027 | 0.033 | 0.412 |
| SGCE_CPG2 | FSWeek | 0.002 | 0.058 | 0.967 |
| SGCE_CPG3 | FSWeek | -0.05 | 0.041 | 0.224 |
| SGCE_CPG4 | FSWeek | -0.015 | 0.031 | 0.616 |
| SGCE_CPG5 | FSWeek | 0.028 | 0.051 | 0.581 |
| SGCE_CPG6 | FSWeek | -0.003 | 0.036 | 0.940 |
| PEG3_CPG1 | FSWeek | -0.003 | 0.032 | 0.933 |
| PEG3_CPG2 | FSWeek | -0.021 | 0.033 | 0.532 |
| PEG3_CPG3 | FSWeek | -0.016 | 0.034 | 0.648 |
| PEG3_CPG4 | FSWeek | 0.000 | 0.046 | 0.999 |
| PEG3_CPG5 | FSWeek | -0.027 | 0.035 | 0.445 |
| PEG3_CPG6 | FSWeek | 0.039 | 0.051 | 0.446 |
| PEG3_CPG7 | FSWeek | -0.012 | 0.028 | 0.657 |
| PEG3_CPG8 | FSWeek | 0.027 | 0.052 | 0.608 |
| PEG3_CPG9 | FSWeek | -0.015 | 0.026 | 0.571 |

| | | | | |
|---|---|---|---|---|
| PEG3_CPG10 | FSWeek | 0.005 | 0.026 | 0.835 |
| PLAGL1_CPG1 | FSWeek | -0.018 | 0.048 | 0.711 |
| PLAGL1_CPG2 | FSWeek | 0.002 | 0.050 | 0.970 |
| PLAGL1_CPG3 | FSWeek | -0.045 | 0.042 | 0.289 |
| PLAGL1_CPG4 | FSWeek | -0.001 | 0.049 | 0.978 |
| PLAGL1_CPG5 | FSWeek | 0.006 | 0.049 | 0.897 |
| PLAGL1_CPG6 | FSWeek | 0.010 | 0.051 | 0.841 |
| NDN_R2bio_CPG1 | FSWeek | -0.006 | 0.032 | 0.843 |
| NDN_R2bio_CPG2 | FSWeek | 0.019 | 0.048 | 0.682 |
| NDN_R2bio_CPG3 | FSWeek | 0.001 | 0.04 | 0.978 |
| NDN_R2bio_CPG4 | FSWeek | 0.008 | 0.038 | 0.836 |
| NDN_R2bio_CPG5 | FSWeek | 0.002 | 0.048 | 0.960 |
| NDN_R2bio_CPG6 | FSWeek | -0.006 | 0.034 | 0.855 |
| SNRPN_CPG1 | FSWeek | -0.005 | 0.019 | 0.783 |
| SNRPN_CPG2 | FSWeek | -0.011 | 0.024 | 0.645 |
| SNRPN_CPG3 | FSWeek | -0.038 | 0.025 | 0.137 |
| SNRPN_CPG4 | FSWeek | 0.044 | 0.048 | 0.360 |
| NNAT_CPG1 | FSWeek | -0.03 | 0.064 | 0.640 |
| NNAT_CPG2 | FSWeek | 0.034 | 0.044 | 0.438 |
| NNAT_CPG3 | FSWeek | -0.028 | 0.039 | 0.479 |
| H19CBS1_CPG1 | FVWeek | 0.006 | 0.008 | 0.443 |
| H19CBS1_CPG2 | FVWeek | 0.000 | 0.005 | 0.974 |
| H19CBS1_CPG3 | FVWeek | 0.004 | 0.005 | 0.401 |
| H19CBS1_CPG4 | FVWeek | 0.003 | 0.006 | 0.602 |
| MEG3CBS1_CPG1 | FVWeek | 0.003 | 0.019 | 0.891 |
| MEG3CBS1_CPG2 | FVWeek | -0.027 | 0.013 | 0.050 |
| MEG3CBS1_CPG3 | FVWeek | 0.000 | 0.017 | 0.997 |
| MEG3CBS1_CPG4 | FVWeek | -0.01 | 0.011 | 0.369 |
| MEG3CBS1_CPG5 | FVWeek | -0.02 | 0.018 | 0.267 |
| MEG3CBS1_CPG6 | FVWeek | -0.047 | 0.017 | 0.066 |
| MEG3CBS1_CPG7 | FVWeek | -0.011 | 0.012 | 0.379 |
| MEG3CBS1_CPG8 | FVWeek | -0.008 | 0.014 | 0.578 |
| MEG3IG_CPG2 | FVWeek | 0.002 | 0.003 | 0.457 |
| MEG3IG_CPG3 | FVWeek | -0.001 | 0.002 | 0.469 |
| MEG3IG_CPG4 | FVWeek | 0.002 | 0.004 | 0.696 |
| MEG3IG_CPG5 | FVWeek | 0.001 | 0.003 | 0.621 |
| MESTIT1_CPG1 | FVWeek | 0.011 | 0.019 | 0.548 |
| MESTIT1_CPG2 | FVWeek | 0.007 | 0.011 | 0.531 |
| MESTIT1_CPG3 | FVWeek | -0.002 | 0.008 | 0.762 |
| MESTIT1_CPG4 | FVWeek | 0.024 | 0.014 | 0.094 |
| DMRO_CPG1 | FVWeek | 0.002 | 0.003 | 0.407 |
| DMRO_CPG2 | FVWeek | -0.006 | 0.007 | 0.392 |
| DMRO_CPG3 | FVWeek | 0.002 | 0.015 | 0.912 |
| GRB10_CPG1 | FVWeek | -0.001 | 0.018 | 0.949 |
| GRB10_CPG2 | FVWeek | -0.004 | 0.005 | 0.475 |
| GRB10_CPG3 | FVWeek | -0.011 | 0.014 | 0.423 |

| | | | | |
|---|---|---|---|---|
| GRB10_CPG4 | FVWeek | 0.003 | 0.017 | 0.863 |
| GRB10_CPG5 | FVWeek | -0.004 | 0.005 | 0.429 |
| GRB10_CPG6 | FVWeek | -0.005 | 0.006 | 0.365 |
| SGCE_CPG1 | FVWeek | -0.014 | 0.011 | 0.199 |
| SGCE_CPG2 | FVWeek | -0.001 | 0.019 | 0.938 |
| SGCE_CPG3 | FVWeek | -0.012 | 0.013 | 0.354 |
| SGCE_CPG4 | FVWeek | -0.019 | 0.011 | 0.073 |
| SGCE_CPG5 | FVWeek | -0.048 | 0.022 | 0.054 |
| SGCE_CPG6 | FVWeek | -0.01 | 0.012 | 0.415 |
| PEG3_CPG1 | FVWeek | -0.011 | 0.011 | 0.301 |
| PEG3_CPG2 | FVWeek | -0.012 | 0.011 | 0.256 |
| PEG3_CPG3 | FVWeek | -0.013 | 0.011 | 0.268 |
| PEG3_CPG4 | FVWeek | -0.032 | 0.016 | 0.051 |
| PEG3_CPG5 | FVWeek | -0.011 | 0.011 | 0.357 |
| PEG3_CPG6 | FVWeek | 0.003 | 0.017 | 0.877 |
| PEG3_CPG7 | FVWeek | -0.015 | 0.009 | 0.118 |
| PEG3_CPG8 | FVWeek | -0.024 | 0.018 | 0.177 |
| PEG3_CPG9 | FVWeek | 0.001 | 0.008 | 0.226 |
| PEG3_CPG10 | FVWeek | -0.007 | 0.009 | 0.432 |
| PLAGL1_CPG1 | FVWeek | -0.013 | 0.016 | 0.403 |
| PLAGL1_CPG2 | FVWeek | -0.01 | 0.017 | 0.553 |
| PLAGL1_CPG3 | FVWeek | -0.018 | 0.014 | 0.194 |
| PLAGL1_CPG4 | FVWeek | -0.01 | 0.017 | 0.554 |
| PLAGL1_CPG5 | FVWeek | -0.009 | 0.017 | 0.576 |
| PLAGL1_CPG6 | FVWeek | -0.006 | 0.017 | 0.703 |
| NDN_R2bio_CPG1 | FVWeek | -0.02 | 0.011 | 0.064 |
| NDN_R2bio_CPG2 | FVWeek | -0.004 | 0.015 | 0.820 |
| NDN_R2bio_CPG3 | FVWeek | -0.006 | 0.014 | 0.649 |
| NDN_R2bio_CPG4 | FVWeek | -0.019 | 0.014 | 0.159 |
| NDN_R2bio_CPG5 | FVWeek | -0.026 | 0.017 | 0.125 |
| NDN_R2bio_CPG6 | FVWeek | -0.005 | 0.012 | 0.697 |
| SNRPN_CPG1 | FVWeek | -0.006 | 0.006 | 0.346 |
| SNRPN_CPG2 | FVWeek | -0.007 | 0.008 | 0.389 |
| SNRPN_CPG3 | FVWeek | -0.009 | 0.008 | 0.255 |
| SNRPN_CPG4 | FVWeek | -0.032 | 0.018 | 0.077 |
| NNAT_CPG1 | FVWeek | -0.019 | 0.02 | 0.345 |
| NNAT_CPG2 | FVWeek | 0.01 | 0.014 | 0.506 |
| **NNAT_CPG3** | **FVWeek** | **-0.061** | **0.014** | **0.000** |
| H19CBS1_CPG1 | FWWeek | -0.004 | 0.005 | 0.430 |
| H19CBS1_CPG2 | FWWeek | -0.003 | 0.003 | 0.221 |
| H19CBS1_CPG3 | FWWeek | -0.002 | 0.003 | 0.520 |
| H19CBS1_CPG4 | FWWeek | 0.000 | 0.004 | 0.960 |
| MEG3CBS1_CPG1 | FWWeek | 0.001 | 0.012 | 0.935 |
| MEG3CBS1_CPG2 | FWWeek | -0.004 | 0.008 | 0.625 |
| MEG3CBS1_CPG3 | FWWeek | -0.006 | 0.011 | 0.587 |
| MEG3CBS1_CPG4 | FWWeek | -0.002 | 0.007 | 0.738 |

| | | | | |
|---|---|---|---|---|
| MEG3CBS1_CPG5 | FWWeek | -0.001 | 0.011 | 0.892 |
| MEG3CBS1_CPG6 | FWWeek | -0.007 | 0.010 | 0.486 |
| MEG3CBS1_CPG7 | FWWeek | -0.002 | 0.008 | 0.815 |
| MEG3CBS1_CPG8 | FWWeek | -0.004 | 0.009 | 0.611 |
| MEG3IG_CPG2 | FWWeek | -0.001 | 0.002 | 0.639 |
| **MEG3IG_CPG3** | **FWWeek** | **-0.003** | **0.001** | **0.026** |
| MEG3IG_CPG4 | FWWeek | 0.005 | 0.003 | 0.089 |
| MEG3IG_CPG5 | FWWeek | 0.000 | 0.002 | 0.804 |
| MESTIT1_CPG1 | FWWeek | -0.025 | 0.012 | 0.053 |
| MESTIT1_CPG2 | FWWeek | 0.018 | 0.006 | 0.058 |
| MESTIT1_CPG3 | FWWeek | -0.002 | 0.005 | 0.741 |
| MESTIT1_CPG4 | FWWeek | 0.017 | 0.009 | 0.057 |
| DMRO_CPG1 | FWWeek | -0.001 | 0.002 | 0.567 |
| **DMRO_CPG2** | **FWWeek** | **-0.010** | **0.004** | **0.021** |
| DMRO_CPG3 | FWWeek | -0.019 | 0.010 | 0.065 |
| GRB10_CPG1 | FWWeek | 0.034 | 0.012 | 0.054 |
| GRB10_CPG2 | FWWeek | 0.001 | 0.003 | 0.755 |
| GRB10_CPG3 | FWWeek | 0.012 | 0.008 | 0.155 |
| GRB10_CPG4 | FWWeek | 0.016 | 0.01 | 0.114 |
| GRB10_CPG5 | FWWeek | 0.003 | 0.003 | 0.409 |
| GRB10_CPG6 | FWWeek | 0.003 | 0.004 | 0.477 |
| SGCE_CPG1 | FWWeek | -0.002 | 0.007 | 0.799 |
| SGCE_CPG2 | FWWeek | -0.014 | 0.012 | 0.233 |
| SGCE_CPG3 | FWWeek | -0.009 | 0.008 | 0.285 |
| SGCE_CPG4 | FWWeek | -0.007 | 0.007 | 0.274 |
| SGCE_CPG5 | FWWeek | -0.028 | 0.012 | 0.051 |
| SGCE_CPG6 | FWWeek | -0.015 | 0.008 | 0.071 |
| PEG3_CPG1 | FWWeek | -0.001 | 0.007 | 0.852 |
| PEG3_CPG2 | FWWeek | -0.002 | 0.007 | 0.768 |
| PEG3_CPG3 | FWWeek | -0.001 | 0.007 | 0.911 |
| PEG3_CPG4 | FWWeek | -0.008 | 0.01 | 0.392 |
| PEG3_CPG5 | FWWeek | -0.001 | 0.007 | 0.861 |
| PEG3_CPG6 | FWWeek | -0.016 | 0.011 | 0.151 |
| PEG3_CPG7 | FWWeek | -0.002 | 0.006 | 0.745 |
| PEG3_CPG8 | FWWeek | 0.022 | 0.011 | 0.168 |
| PEG3_CPG9 | FWWeek | 0.000 | 0.005 | 0.926 |
| PEG3_CPG10 | FWWeek | -0.002 | 0.006 | 0.724 |
| PLAGL1_CPG1 | FWWeek | 0.001 | 0.01 | 0.894 |
| PLAGL1_CPG2 | FWWeek | -0.009 | 0.011 | 0.404 |
| PLAGL1_CPG3 | FWWeek | -0.004 | 0.009 | 0.616 |
| PLAGL1_CPG4 | FWWeek | 0.001 | 0.011 | 0.968 |
| PLAGL1_CPG5 | FWWeek | -0.009 | 0.011 | 0.383 |
| PLAGL1_CPG6 | FWWeek | -0.005 | 0.011 | 0.612 |
| NDN_R2bio_CPG1 | FWWeek | -0.007 | 0.007 | 0.294 |
| NDN_R2bio_CPG2 | FWWeek | 0.012 | 0.011 | 0.215 |
| NDN_R2bio_CPG3 | FWWeek | -0.023 | 0.009 | 0.054 |

| | | | | |
|---|---|---|---|---|
| NDN_R2bio_CPG4 | FWWeek | -0.013 | 0.009 | 0.122 |
| NDN_R2bio_CPG5 | FWWeek | -0.008 | 0.012 | 0.442 |
| NDN_R2bio_CPG6 | FWWeek | 0.001 | 0.008 | 0.929 |
| SNRPN_CPG1 | FWWeek | -0.006 | 0.004 | 0.125 |
| SNRPN_CPG2 | FWWeek | -0.004 | 0.005 | 0.445 |
| SNRPN_CPG3 | FWWeek | 0.005 | 0.005 | 0.272 |
| SNRPN_CPG4 | FWWeek | -0.009 | 0.011 | 0.434 |
| NNAT_CPG1 | FWWeek | -0.007 | 0.013 | 0.591 |
| NNAT_CPG2 | FWWeek | 0.003 | 0.009 | 0.771 |
| NNAT_CPG3 | FWWeek | 0.01 | 0.007 | 0.149 |
