# Supplementary material for: Opposing Epigenetic Signatures in Human Sperm by Intake of Fast Food Versus Healthy Food
Source: Front Endocrinol (Lausanne). 2021 Apr 23;12:625204. doi: 10.3389/fendo.2021.625204 (PMC8103543; doi:10.3389/fendo.2021.625204)
Supplement: Supplementary file 8 [file DataSheet_8.pdf]

Supplementary Table 5: Associations between all Dietary Items and the Mean DNA Methylation Level at Imprinted Genes

The mean of the CpGs for each of the 12 DMRs were considered; TIEGER data (n=67). Beta regression models were fitted for each DMR after adjusting for age, BMI, and patient status. In bold: significant results ($p<0.05$). Beta-coefficients are provided in log-scale (outliers were reconsidered). DMRO refers to the DMR of *IGF2*. Food items per week are shown as follows: FBWEEK (for burgers), FFWEEK (for fruits/nuts), FMWEEK (for meat), FPWEEK (for pizza), FRWEEK (for fries), FSWEEK (for fish/seafood), FVWEEK (for vegetables/salads/soups), FWWEEK (for whole grain bread/flakes).

Gene	Diet	Beta	SE	p-value
MEAN_H19CBS1_CPGs	FBWeek	0.003	0.012	0.811
MEAN_MEG3CBS1_CPGs	FBWeek	0.018	0.049	0.713
MEAN_MEG3IG_CPGs	FBWeek	0.001	0.008	0.853
MEAN_MESTIT1_CPGs	FBWeek	0.020	0.020	0.310
MEAN_DMRO_CPGs	FBWeek	-0.001	0.019	0.957
MEAN_GRB10_CPGs	FBWeek	-0.007	0.026	0.776
MEAN_SGCE_CPGs	FBWeek	-0.013	0.046	0.771
MEAN_PEG3_CPGs	FBWeek	-0.001	0.039	0.982
MEAN_PLAGL1_CPGs	FBWeek	-0.014	0.056	0.803
MEAN_NDN_R2bio_CPGs	FBWeek	0.001	0.040	0.973
MEAN_SNRPN_CPGs	FBWeek	-0.001	0.028	0.975
MEAN_NNAT_CPGs	FBWeek	-0.042	0.043	0.323
MEAN_H19CBS1_CPGs	FFWeek	0.001	0.004	0.923
MEAN_MEG3CBS1_CPGs	FFWeek	-0.008	0.014	0.589
MEAN_MEG3IG_CPGs	FFWeek	-0.001	0.002	0.512
MEAN_MESTIT1_CPGs	FFWeek	0.007	0.007	0.291
MEAN_DMRO_CPGs	FFWeek	0.001	0.005	0.785
MEAN_GRB10_CPGs	FFWeek	0.001	0.007	0.942
MEAN_SGCE_CPGs	FFWeek	-0.011	0.012	0.382
MEAN_PEG3_CPGs	FFWeek	0.000	0.011	0.993
MEAN_PLAGL1_CPGs	FFWeek	-0.006	0.016	0.706
MEAN_NDN_R2bio_CPGs	FFWeek	-0.008	0.011	0.466
MEAN_SNRPN_CPGs	FFWeek	-0.003	0.008	0.667
MEAN_NNAT_CPGs	FFWeek	-0.014	0.011	0.242
MEAN_H19CBS1_CPGs	FMWeek	0.001	0.004	0.766
MEAN_MEG3CBS1_CPGs	FMWeek	-0.007	0.014	0.601
MEAN_MEG3IG_CPGs	FMWeek	0.000	0.002	0.907
MEAN_MESTIT1_CPGs	FMWeek	0.001	0.006	0.952
MEAN_DMRO_CPGs	FMWeek	-0.005	0.005	0.405
MEAN_GRB10_CPGs	FMWeek	-0.006	0.007	0.368
MEAN_SGCE_CPGs	FMWeek	-0.005	0.012	0.714
MEAN_PEG3_CPGs	FMWeek	-0.005	0.011	0.661
MEAN_PLAGL1_CPGs	FMWeek	0.001	0.016	0.967
MEAN_NDN_R2bio_CPGs	FMWeek	-0.015	0.011	0.175
MEAN_SNRPN_CPGs	FMWeek	-0.002	0.008	0.839
MEAN_NNAT_CPGs	FMWeek	-0.008	0.011	0.490
MEAN_H19CBS1_CPGs	FPWeek	0.013	0.01	0.182
MEAN_MEG3CBS1_CPGs	FPWeek	-0.029	0.038	0.440
MEAN_MEG3IG_CPGs	FPWeek	0.006	0.006	0.281
MEAN_MESTIT1_CPGs	FPWeek	-0.003	0.019	0.803
MEAN_DMRO_CPGs	FPWeek	0.032	0.015	0.036
MEAN_GRB10_CPGs	FPWeek	0.011	0.019	0.572

MEAN_SGCE_CPGs	FPWeek	-0.026	0.033	0.431
MEAN_PEG3_CPGs	FPWeek	-0.015	0.03	0.623
MEAN_PLAGL1_CPGs	FPWeek	-0.045	0.044	0.297
MEAN_NDN_R2bio_CPGs	FPWeek	-0.003	0.031	0.914
MEAN_SNRPN_CPGs	FPWeek	-0.019	0.021	0.361
MEAN_NNAT_CPGs	FPWeek	0.014	0.029	0.616
MEAN_H19CBS1_CPGs	FRWeek	0.021	0.016	0.178
MEAN_MEG3CBS1_CPGs	FRWeek	0.036	0.061	0.561
MEAN_MEG3IG_CPGs	FRWeek	0.019	0.010	0.048
MEAN_MESTIT1_CPGs	FRWeek	0.018	0.029	0.529
MEAN_DMRO_CPGs	FRWeek	0.053	0.024	0.033
MEAN_GRB10_CPGs	FRWeek	0.020	0.031	0.518
MEAN_SGCE_CPGs	FRWeek	0.008	0.056	0.887
MEAN_PEG3_CPGs	FRWeek	0.029	0.048	0.547
MEAN_PLAGL1_CPGs	FRWeek	0.038	0.072	0.594
MEAN_NDN_R2bio_CPGs	FRWeek	0.037	0.051	0.470
MEAN_SNRPN_CPGs	FRWeek	0.024	0.035	0.492
MEAN_NNAT_CPGs	FRWeek	0.041	0.047	0.388
MEAN_H19CBS1_CPGs	FSWeek	-0.005	0.011	0.668
MEAN_MEG3CBS1_CPGs	FSWeek	-0.032	0.041	0.446
MEAN_MEG3IG_CPGs	FSWeek	-0.001	0.007	0.827
MEAN_MESTIT1_CPGs	FSWeek	0.039	0.022	0.130
MEAN_DMRO_CPGs	FSWeek	0.014	0.017	0.412
MEAN_GRB10_CPGs	FSWeek	-0.017	0.021	0.427
MEAN_SGCE_CPGs	FSWeek	-0.024	0.035	0.498
MEAN_PEG3_CPGs	FSWeek	-0.012	0.032	0.713
MEAN_PLAGL1_CPGs	FSWeek	-0.020	0.046	0.660
MEAN_NDN_R2bio_CPGs	FSWeek	-0.005	0.032	0.868
MEAN_SNRPN_CPGs	FSWeek	-0.011	0.022	0.630
MEAN_NNAT_CPGs	FSWeek	-0.019	0.033	0.575
MEAN_H19CBS1_CPGs	FVWeek	0.002	0.003	0.629
MEAN_MEG3CBS1_CPGs	FVWeek	-0.014	0.014	0.299
MEAN_MEG3IG_CPGs	FVWeek	0.001	0.002	0.636
MEAN_MESTIT1_CPGs	FVWeek	0.004	0.007	0.571
MEAN_DMRO_CPGs	FVWeek	0.002	0.005	0.765
MEAN_GRB10_CPGs	FVWeek	-0.007	0.007	0.283
MEAN_SGCE_CPGs	FVWeek	-0.018	0.012	0.129
MEAN_PEG3_CPGs	FVWeek	-0.013	0.011	0.246
MEAN_PLAGL1_CPGs	FVWeek	-0.016	0.016	0.311
MEAN_NDN_R2bio_CPGs	FVWeek	-0.016	0.011	0.146
MEAN_SNRPN_CPGs	FVWeek	-0.009	0.008	0.249
MEAN_NNAT_CPGs	FVWeek	-0.026	0.011	0.029
MEAN_H19CBS1_CPGs	FWWeek	-0.003	0.002	0.208
MEAN_MEG3CBS1_CPGs	FWWeek	-0.003	0.009	0.749
MEAN_MEG3IG_CPGs	FWWeek	0.000	0.001	0.931
MEAN_MESTIT1_CPGs	FWWeek	-0.001	0.004	0.903

MEAN_DMRO_CPGs	FWWeek	-0.006	0.003	0.076
MEAN_GRB10_CPGs	FWWeek	0.004	0.004	0.320
MEAN_SGCE_CPGs	FWWeek	-0.008	0.008	0.294
MEAN_PEG3_CPGs	FWWeek	-0.003	0.007	0.702
MEAN_PLAGL1_CPGs	FWWeek	-0.002	0.010	0.814
MEAN_NDN_R2bio_CPGs	FWWeek	-0.005	0.007	0.440
MEAN_SNRPN_CPGs	FWWeek	-0.006	0.005	0.241
MEAN_NNAT_CPGs	FWWeek	0.003	0.007	0.639
