# Supplementary material for: Opposing Epigenetic Signatures in Human Sperm by Intake of Fast Food Versus Healthy Food
Source: Front Endocrinol (Lausanne). 2021 Apr 23;12:625204. doi: 10.3389/fendo.2021.625204 (PMC8103543; doi:10.3389/fendo.2021.625204)
Supplement: Supplementary file 9 [file DataSheet_9.pdf]

frontiers

Supplementary Table 6: Chromosome Number and Position of all CpGs Studied

DMR CpG positions of studied imprinted genes according to UCSC Genome Browser hg38 (https://genome.ucsc.edu/cgi-bin/hgGateway). In parentheses: probe IDs of the Infinium HumanMethylation450 BeadChips if our sites overlap the Illumina platform. TSS: Transcription Start Sites; note, in some cases there is more than one TSS, and imprinted genes also act *in trans* (on the expression of other imprinted genes).

DMR	Chr.	TSS	CpG1	CpG2	CpG3	CpG4	CpG5	CpG6	CpG7	CpG8	CpG9	CpG10
**GRB10**	**7**	50.793.453;<br>50.792.874;<br>50.782.896;<br>50.732.353;<br>50.705.301	50.782.914	50.782.916	50.782.922	50.782.930	50.782.934	50.782.937				
**H19**	**11**	2.001.466;<br>1.997.875	2.003.023	2.003.026	2.003.028	2.003.030						
**IGF2**	**11**	2.149.603;<br>2.140.943;<br>2.139.389;<br>2.137.288	2.148.273	2.148.285	2.148.288							
**MEG3**	**14**	100.826.108	100.827.977	100.827.990	100.827.999	100.828.008	100.828.012	100.828.019	100.828.024	100.828.027		
**MEG3-IG**	**14**	100.826.108		100.810.928	100.810.930	100.810.946	100.810.948					
**MEST**	**7**	130.486.175;<br>130.486.347;<br>130.491.329;<br>130.492.058	130.492.539	130.492.571<br>(cg09462536)	130.492.577<br>(cg22592140)	130.492.580<br>(cg03588221)						
**NDN**	**15**	23.687.305	23.686.580	23.686.589	23.686.592	23.686.604	23.686.607	23.686.609				
**NNAT**	**20**	37.521.250	37.520.954	37.520.985	37.521.007							
**PEG3**	**19**	56.840.726;<br>56.837.808	56.840.604	56.840.610	56.840.618	56.840.625	56.840.638	56.840.642	56.840.645<br>(cg15777825)	56.840.649<br>(cg10204755)	56.840.652<br>(cg12205903)	56.840.655
**PLAGL1**	**6**	144.064.599;<br>144.055.262;<br>144.008.259	144.008.041	144.008.055	144.008.059	144.008.062	144.008.068	144.008.070				
**SGCE/PEG10**	**7**	94.656.133;<br>94.669.694	94.657.883<br>(cg12397924)	94.657.898<br>(cg15696408)	94.657.911	94.657.913	94.657.927	94.657.929<br>(cg22820921)				
**SNRPN**	**15**	25.419.461;<br>24.856.571;<br>24.954.987	24.955.258	24.955.262	24.955.272	24.955.274						
